# Anti-proliferative and pro-apoptotic activity of glycosidic derivatives of lawsone in melanoma cancer cell

**DOI:** 10.1186/s12885-021-08404-4

**Published:** 2021-06-02

**Authors:** Mariana Nobre Farias de Franca, Raquel Geralda Isidório, João Henrique Oliveira Bonifacio, Edmilson Willian Propheta dos Santos, Jileno Ferreira Santos, Flaviano Melo Ottoni, Waldecy de Lucca Junior, Ricardo Scher, Ricardo José Alves, Cristiane Bani Corrêa

**Affiliations:** 1 Laboratory of Biology and Immunology of Cancer and Leishmania, Department of Morphology, Federal University of Sergipe, São Cristóvão, Sergipe Brasil; 2 Graduate program in health sciences, Federal University of Sergipe, Aracaju, Sergipe, Brazil; 3 Laboratory of Pharmaceutical Chemistry, Department of Pharmaceutical Products, Faculty of Pharmacy, Federal University of Minas Gerais, Belo Horizonte, Minas Gerais Brazil; 4 Laboratory of Molecular Neuroscience of Sergipe, Department of Morphology, Federal University of Sergipe, São Cristóvão, Sergipe Brazil

**Keywords:** Lawsone, Melanoma, Cytotoxicity, Apoptosis, Flow cytometer, Migration, Clonogenic assay

## Abstract

**Background:**

Melanoma is a malignant cancer that affects melanocytes and is considered the most aggressive skin-type cancer. The prevalence for melanoma cancer for the last five year is about one million cases. The impact caused of this and other types of cancer, revel the importance of research into potential active compounds. The natural products are an important source of compounds with biological activity and research with natural products may enable the discovery of compounds with potential activity in tumor cells.

**Methods:**

The Sulforhodamine B was used to determine cell density after treatment with lawsone derivatives. Apoptosis and necrosis were analyzed by flow cytometer. Morphological changes were observed by fluorescence using the Phalloidin/FITC and DAPI stains. The clonogenic and wound healing assays were used to analyze reduction of colonies formation and migratory capacity of melanoma cells, respectability.

**Results:**

In pharmacological screening, seven compounds derived from lawsone were considered to have high cytotoxic activity (GI > 75%). Three compounds were selected to assess the inhibitory concentration for 50% of cells (IC_50_), and the compound 9, that has IC_50_ 5.3 μM in melanoma cells, was selected for further analyses in this cell line. The clonogenic assay showed that the compound is capable of reducing the formation of melanoma colonies at 10.6 μM concentration. The compound induced apoptotic morphological changes in melanoma cells and increased by 50% the cells dying from apoptosis. Also, this compound reduced the migratory capacity of melanoma cells.

**Conclusions:**

The results of this study showed that the evaluated lawsone derivatives have potential activity on tumor cells. The compound 9 is capable of inducing cell death by apoptosis in melanoma cells (B16F10).

**Supplementary Information:**

The online version contains supplementary material available at 10.1186/s12885-021-08404-4.

## Introduction

Cancer is a non-transmissible chronic disease that affects more than 50,5 million people in the world [1]. It has the second highest mortality rate, surpassed only by cardiovascular diseases. It is considered a serious public health matter responsible for about 9.9 million deaths in 2020, representing 1 in 8 and 1 in 11 deaths for men and woman, respectively [2, 3].

Melanoma is the most lethal among the skin type cancers [4]. Data on the incidence of cutaneous melanoma show that more than 300 thousand people were affected by the disease in 2020, in the last five years, there was about one million cases [5]. The risk factors associated with the development of cutaneous melanoma are linked with environmental factors, such as prolonged exposure to ultraviolet radiation, and genetic predisposition [4].

The global impact caused by the high incidence rates and prevalence of cancer, associated with the heterogeneity of the disease, which presents different forms and specificities and the chemoresistance developed by tumor cells to antineoplastic treatment reveal the importance of the search for new bioactive compounds with potential antitumor activity [6]. Natural products are an important source of compounds with biological activity against several diseases and they may also prove to be less harmful and toxic [7]. As an example, the studies with α-mangustin, an xanthone showed promising in vitro results for the reduction of melanin production and can be a promising cosmetic compound [8]. In the context of cancer, resveratrol a naturally occurring polyphenol that has activity against cancers by acting on several molecular targets. Resveratrol can act, for example, by inhibiting the expression of Programmed death-1 in the T cell membrane. This inhibition prevents the receptor from being bound to the PD-1 L expressed in tumor cells, which would inhibit the cytotoxic activity of T cells [7, 9]. About 40% of the drugs approved for commercialization and 64.9% of the existing antineoplastic drugs are natural products or are derived from natural products [10]. Drugs like doxorubicin and vincristine used in anticancer therapy are examples of drugs that had their origin in animal and plant sources, respectively [11, 12].

The secondary metabolite naphthoquinone is an example of a class of compounds of plant and animal origin that has biological activity on several diseases, including some types of cancers [13]. Lawsone is a naphthoquinone widely studied in terms of its biological activity. This compound, found in *Lawsonia inermis*, is a precursor in the synthesis of compounds with potential biological activity, and many of its derivatives have antitumor, antibacterial and antifungal activity [14, 15].

It is important to think that, even though some chemical compounds do not become a drug used in the treatment of diseases, they can serve as a precursor to new compounds with the potential to generate new drugs [14–16]. For example, the synthesis of atovaquone from the lawsone compound resulted in a compound with antimalarial activity [17]. Besides, changes in the chemical structure of compounds can give better pharmacokinetic profiles to the new synthesized compounds, as well as increased it cytotoxicity to tumor cells and reduce side effects [16, 17]. In this way, we can consider that chemical changes in lawsone compounds can result in newly and improved biologically active compounds [18, 19]. So, considering the hypothesis that synthetic derivatives of lawsone may present potential cytotoxic activity against tumor cells, this study was performed aiming to evaluate the cytotoxic activity of compounds structurally correlated with lawsone in three distinct tumor cell lines.

## Materials and methods

### Chemicals

Dulbeccos’s Modified Eagle Medium (DMEM), Trypsin, Dimethylsulfoxide, DAPI, Sulforhodamine B (SRB), Triton X 100 and Phalloidin-FITC were purchased from Sigma Aldrich (Saint Louis, MO, USA), Fetal Bovine Serum (FBS), antibiotic (penicillin 10,000 U/ml; streptomycin 10,000 mg/ml) and Bovine Serum Albumin (BSA) from Gibco (Life Technologies, India) EDTA, Doxorubicin Hydrochloride (Rubidox, Bergamo), Trichloroacetic acid (Neon), Acetic acid (Synth), Methyl alcohol (Neon), TRIS-base (Inlab Confiança, Brazil), Dead Cell Apoptosis Kit (Life Technology™, Carlsbad, California), Accutase® (Thermo Fisher Scientific, Carlsbad, Califórnia, EUA).

### Lawsone derivatives

Lawsone (2-hydroxy-1,4-naphtoquinone) and sixteen of its derivatives were obtained in partnership with the Laboratory of Pharmaceutical Chemistry, Faculty of Pharmacy, Federal University of Minas Gerais (UFMG). Lawsone was purchased from Sigma-Aldrich Brazil, São Paulo. Compounds 1 [20], 4 [21] and 9–16 [22] were prepared according to literature. Data regarding the synthesis and characterization of compounds 2, 3 and 5–8 are described in Additional file 1. A stock solution of the lawsone derivatives was made by dissolving the compound in dimethylsulfoxide solvent (DMSO) and kept refrigerated at − 4 °C.

### Cell lines

The cell lines of lung carcinoma (A549), melanoma (B16F10) and glioma (C6) were purchased from the Federal University of Rio de Janeiro. The cells were cultured in a humidified incubator at 37 °C in 5% CO_2_ in Dulbeccos’s Modified Eagle Medium (DMEM) medium supplemented with 10% Fetal Bovine Serum (FBS) and 1% antibiotic (10,000 U/mL penicillin; 10,000 mg/mL streptomycin).

### Determination of cell growth inhibition and IC_50_

**Fig. 1:**
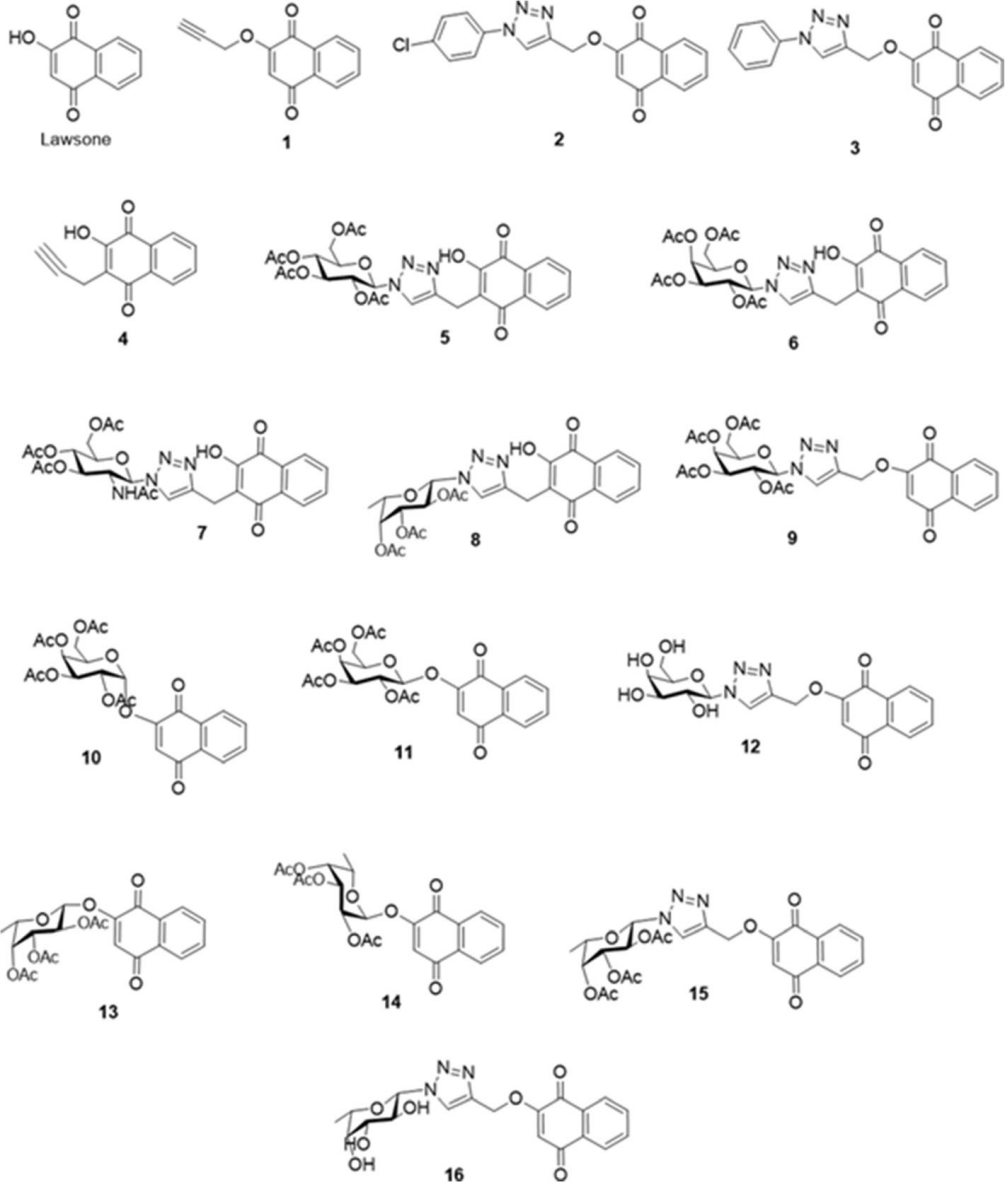
Structure of evaluated compounds

A first screening of the cytotoxic activity of lawsone derivatives (Fig. 1) was done by Sulforhodamine B assay (SRB), following a protocol adapted from [23, 24]. The cell lines A549, B16F10 and C6 were seeded in 96-wells plate at a density of 1 × 10^4^ cells per well for A549 and C6 and 3 × 10^3^ for B16F10 in 200 μL of medium. After 24 h, the cells were treated with lawsone derivatives at a concentration of 25 μM for 72 h. Dimethylsulfoxide (DMSO, 0.05%) was used as vehicle control and Doxorubicin Hydrochloride 10 μM was used was positive control. Three independent experiments were performed in quadruplicate.

After treatment, the supernatant was discarded, and the cells were fixed with 100 μL of trichloroacetic acid 30% for 1 h at 4 °C and stained with 100 μL of SRB (0.057% w/v) for 30 min. Excess dye was removed by washing the plate with 1% acetic acid for 3 times, and intracellular dye was dissolved in 200 μL of TRIS (10 mM; pH 10,5) for 30 min. Absorbance was read in a microplate reader (Synergy H1, Biotek, VT, EUA) at 510 nm. The absorbance was converted in growth inhibition by the following equation:

$$ \%\mathrm{Growth}\ \mathrm{inhibition}=\left\{100-\left[\left(\mathrm{Abs}\ \left(\mathrm{treated}\ \mathrm{cells}\right)/\mathrm{Abs}.\left(\mathrm{vehicle}\ \mathrm{control}\right)\right)\ \mathrm{x}\ 100\right]\right\} $$

The lawsone derivatives that showed the highest growth inhibition were selected to determine the IC_50_ using the same protocol described above. The cell lines A549, B16F10 and C6 were treated with lawsone and the derivatives 9, 10, 11 in concentrations ranging from 0,8 to 25 μM.

### Clonogenic assay

To assess the ability of lawsone derivatives to reduce melanoma colonies, the clonogenic assay was performed following a protocol adapted from [25]. B16F10 cells were seeded in a concentration of 300 cells per well in a 6-well plate. After 24 h the cells were treated with 2.6, 5.3 and 10.6 μM of compound 9 for 72 h at the same conditions. Dimethylsulfoxide (0.02%) and Doxorubicin Hydrochloride (10 μM) were used as vehicle control and positive death control, respectively. After 6 days, the cells were fixed with methanol:acetic acid (3:1) for 5 min and stained with violet crystal 0.02% in water, for 30 min. Colony formation analyzes were performed using ImageJ 1.46 software.

### Wound healing assay

The wound-healing assay was made using the protocol with modifications of Liang and colleagues [26]. B16F10 cells were seeded in 12-well plate at a density of 3 × 10^5^ cells per well. After 24 h, the cell monolayer was scratched with a tip of p200 pipette creating a straight line wound, the debris were removed by washing with PBS and the cells were treated with 2.6, 5.3 and 10.6 μM the derivative 9 for 24 h. Dimethylsulfoxide (0.02%) was used as vehicle control and Doxorubicin Hydrochloride 10 μM was used as positive control. The images were acquired 0, 24 and 48 h of the scratch using a microscopy Olympus. The percentage of wound closure was calculated for each treatment and controls comparing the time points 24 and 48 h with the time point zero, using the equation proposed by [27]. The images were analyzed using ImageJ 1.46 software.

### Cell morphology analysis

B16F10 cells were seeded in a 48-well plate at density of 5 × 10^3^ cell per well. After 24 h, cells were treated with 2.6, 5.3 and 10.6 μM of derivative 9 for 24 h. Dimethylsulfoxide (0.02%) was used as vehicle control and Doxorubicin Hydrochloride 10 μM was used as positive control. After treatment cells were washed with 1x PBS and fixed with 4% formaldehyde for 15 min. Cells were permeabilized with 0.2% Triton X 100 solution for 15 min and 1% Bovine Serum Albumin (BSA) for 30 min. The cytoskeleton was stained with Phalloidin-FITC 25 μg/mL for 30 min and the cell nucleus was stained with DAPI 1 μg / mL for 10 min, in the dark. The images were acquired using a microscopy Olympus, IX81 and the morphological changes of nucleus and cytoplasm, as well as chromatin fragmentation [28].

### Cell death evaluation by Annexin V/PI flow cytometry assay

B16F10 cells were seeded in a 12-well plate ate a density of 5 × 10^4^ cell per well. After 24 h, cells were treated with derivative 9 with the concentrations of 2.6, 5.3 and 10.6 μM for 24 h. Dimethylsulfoxide (0.02%) was used as vehicle control and Doxorubicin Hydrochloride 10 μM was used as positive control. After treatment, the cells were harvested using Accutase®, washed with 1x PBS and stained using Dead Cell Apoptosis Kit following the manufactory instructions. The cells were analyzed in a flow cytometer (Attune NxT Acoustic Focusing Cytometer, Thermo Fisher Scientific).

### Statistical analysis

For all experiments, a 95% confidence interval was used and *p* < 0.05 values considered statistically significant. Analyzes and graphs as well as IC_50_ were obtained using the GraphPad Prism 8 program. Shapiro-Wilk normality test was applied to assess the normal distribution of the data. For comparison between groups, ANOVA was used, followed by Dunnett post-test.

## Results

### Lawsone derivatives reduce the viability of tumor cell lines

Sixteen lawsone derivatives (Fig. 1) were evaluated for their effect in reducing cell growth of three tumor cell lines, A549 (lung carcinoma), B16F10 (melanoma) and C6 (glioma). The compounds were tested by pharmacological screening at a single concentration of 25 μM, for 72 h using the Sulforhodamine B assay.

The compounds were classified according to their potential to inhibit cell proliferation using the following intensity scale: compounds which showed a percentage of growth inhibition (GI) lower than 50% were classified as low cytotoxic activity, between 51 and 75% as intermediate cytotoxic activity and higher than 75% as high cytotoxic activity [29]. The compounds 5, 6, 7, 8, 12 and 16 showed a percentage of growth inhibition lower than 50% in all cell lineage evaluates were classified as having low cytotoxic activity. Other compounds evaluated (2, 3 and 4) showed growth inhibition ranging from low, intermediate and high cytotoxic activity in the cell lines. Seven compounds (1, 9, 10, 11, 13, 14 and 15) showed high cytotoxic activity (GI > 75%) in the three cell lines. Among the seven compounds, six of them (1, 9, 10, 11 and 15) showed GI > 90% for the three cell lines (Table 1).

**Table 1:**
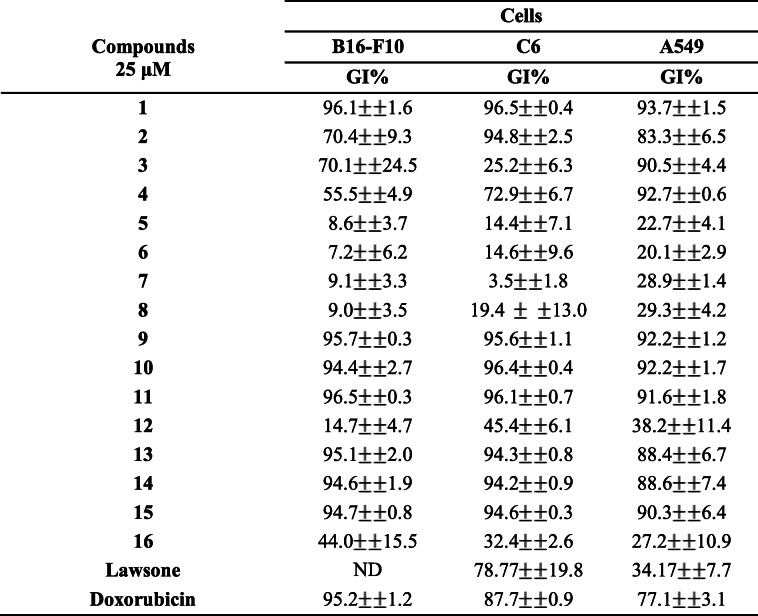
Percentage of growth inhibition in B16F10, C6 and A549 after treatment with synthetic lawsone derivatives

Mean values ± standard deviation of at least three independent experiments after treatments with synthetic derivatives of lawsone for 72 h. Doxorubicin 10 μM was used as a positive control. ND - not determined

The activity of lawsone, compound of origin in the synthesis of the derivatives studied in this research, was also evaluated. It was possible to observe that lawsone showed low cytotoxic activity (GI = 34.17%) in A549 and high cytotoxic activity (GI = 78.7%) in C6. It was not possible to observe cytotoxic activity in B16F10 at a concentration of 25 μM.

Doxorubicin, an important chemotherapeutic with cytotoxic activity on several types of cancer, was used as a positive control to estimate the viability of tumor cells [30]. This drug, as well as the compounds evaluated in this study, is a quinone and has a structure capable of releasing free radicals that cause cellular stress [31]. The drug showed a high growth inhibition in the three tumor lines evaluated (GI 77.1–99.2%) at 10 μM.

**Fig. 2:**
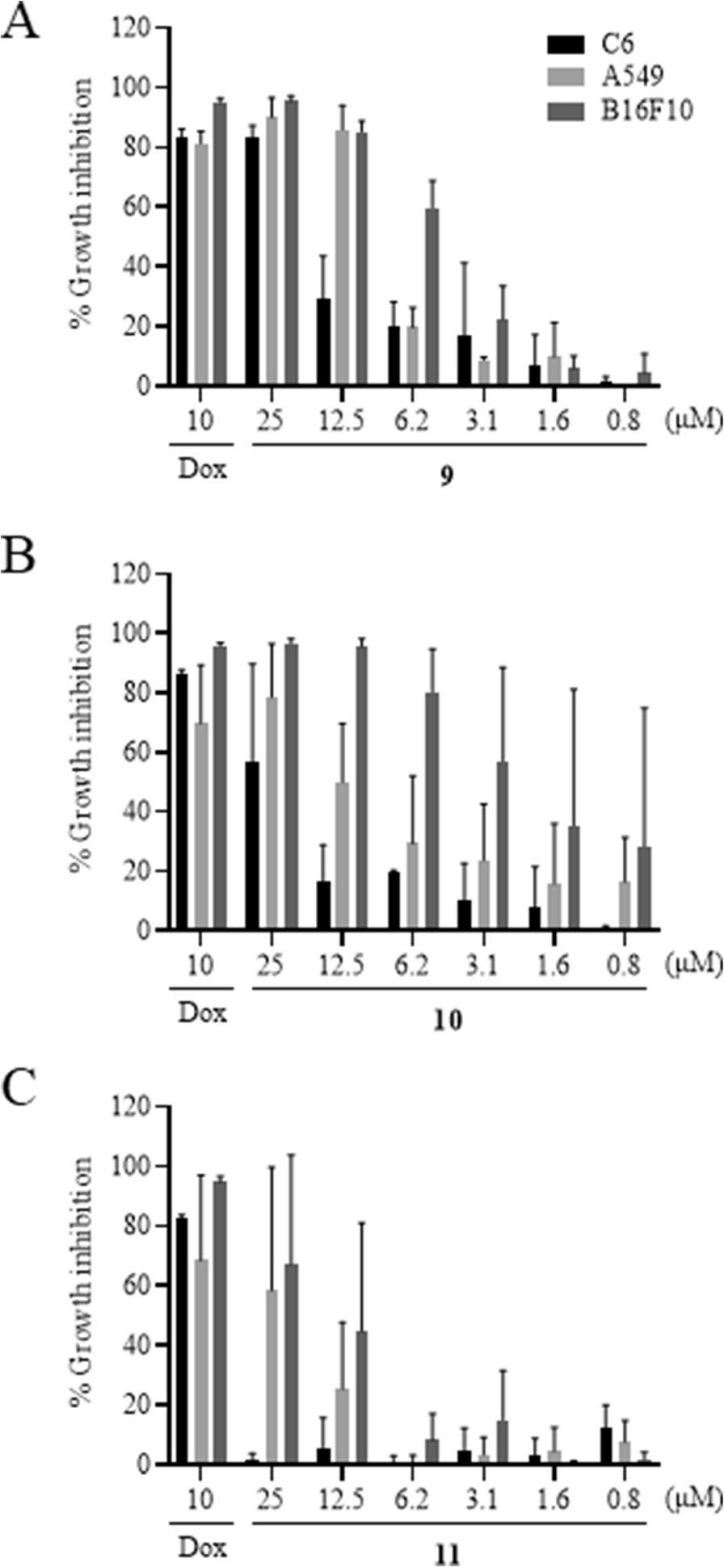
Growth inhibitation of A549, B16F10 and C6 cell lines after treatment with compunds 9 (**A**), 10 (**B**) and 11 (**C**). Values of mean ± standard deviation of at least three independent experiments after treatments with synthetic derivatives of lawsone for 72 h. Doxorubicin 10 μM was used as positive control, respectively

Due to the high cytotoxic activity of compounds 9, 10 and 11 that showed GI > 90% and their structural chemical characteristics, these compounds were selected to determine the maximum concentration that inhibits 50% of cells growth (IC_50_). The growth inhibition profile and the IC_50_ values obtained are presented in Fig. 2 and Table 2, respectively.

**Table 2:** IC_50_ values of compounds 9, 10 and 11 and lawsone against C6, B16-F10 and A549

Compounds	Cells		
	C6	B16F10	A549
	CI_50_ (μM)	CI_50_ (μM)	CI_50_ (μM)
**9**	14.7 (10.3–21.4)	5.3 (4.8–5.8)	8.5 (7.6–9.5)
**10**	24.0 (16.5–80.1)	2.2 (1.0–4.0)	10.6 (6.9–18.1)
**11**	ND	15.7 (10.6–29.5)	21.2 (15.6–38.5)
**Lawsone**	26.8 (17.1–40.0)	ND	> 50

Values expressed as mean and 95% confidence interval of at least three independent experiments after treatment with synthetic derivatives of lawsone and lawsone for 72 h. ND - not determined

The IC_50_ values expressed in Table 2 show that, compared with lawsone, the three synthetic derivatives analyzed showed to be more cytotoxic to cell lines. Among them, the compound 9 and 10 showed the highest cytotoxic activity, both in B16F10. When compared with lawsone, the three synthetic derivatives analyzed showed to be more cytotoxic to cell lines. Because of its low concentration of IC_50_ of compound 9 on three cell lines, and the fact of melanoma has the lowest IC_50_ for this compound, as well as the severity of the melanoma and the importance of studies with potential antitumor compounds for highly lethal cancer, this compound was selected for the next assays in B16F10.

### Compounds 9 reduces the ability of tumor cells to form clones and colonies

**Fig. 3:**
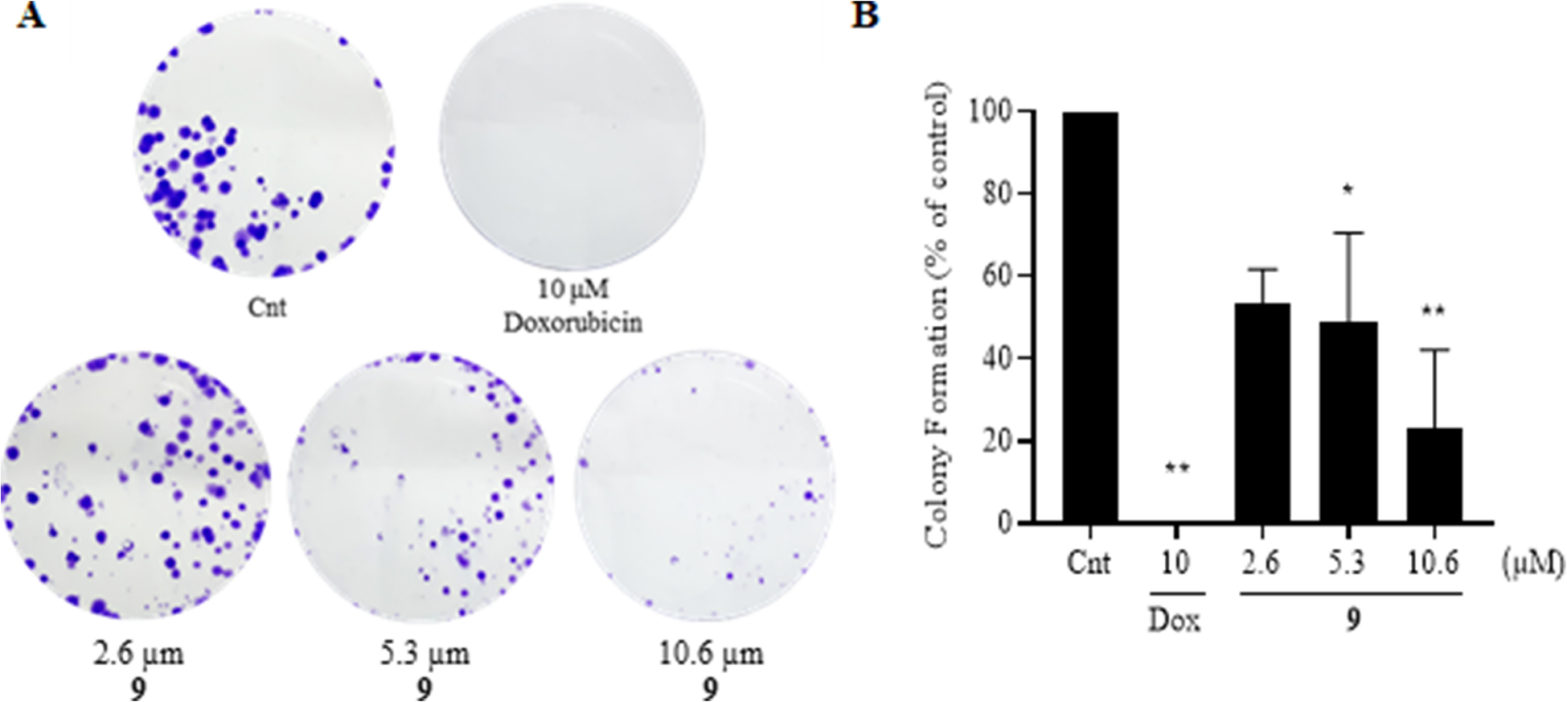
Inhibitory effect of molecule 9 on the formation of B16F10 cells colonies. **A** Representative experiment showing colonies formed after treatment with 2.6, 5.3 and 10.6 μM of compound 9 and 10 days of growth **B** Percentage of colony formation after treatment relative control. Dox = doxorubicin positive control. Mean ± SD obtained from two independent experiments. The analysis of difference between treatments and control was performed by ANOVA, followed by Dunnett post-test (*) *p* < 0.05, (**) *p* < 0.01

The clonogenic assay was performed to evaluate the ability of a single melanoma cell (B16F10) to form clones and colonies after treatment with 2.6, 5.3 and 10.6 μM of compound 9 for 72 h. These concentrations were defined based on the IC_50_ value and are equivalent to half and twice the IC_50_ value. A reduction in the number of colonies derived from cells treated with the two highest concentrations (5.3 and 10.6 μM) was evident, as showed in the Fig. 3A.

The analyzes of the results obtained from two independent experiments (Fig. 3B), revealed that the treatment with the highest concentration of compound 9 (10.6 μM) reduced the capacity of colony formation by approximately 75% compared to negative control. Doxorubicin reduced the capacity of melanoma cells to form clones and colonies by 100% at a concentration of 10 μM.

### The cells of the tumor line B16F10 show reduced migration when exposed to compound 9

**Fig. 4:**
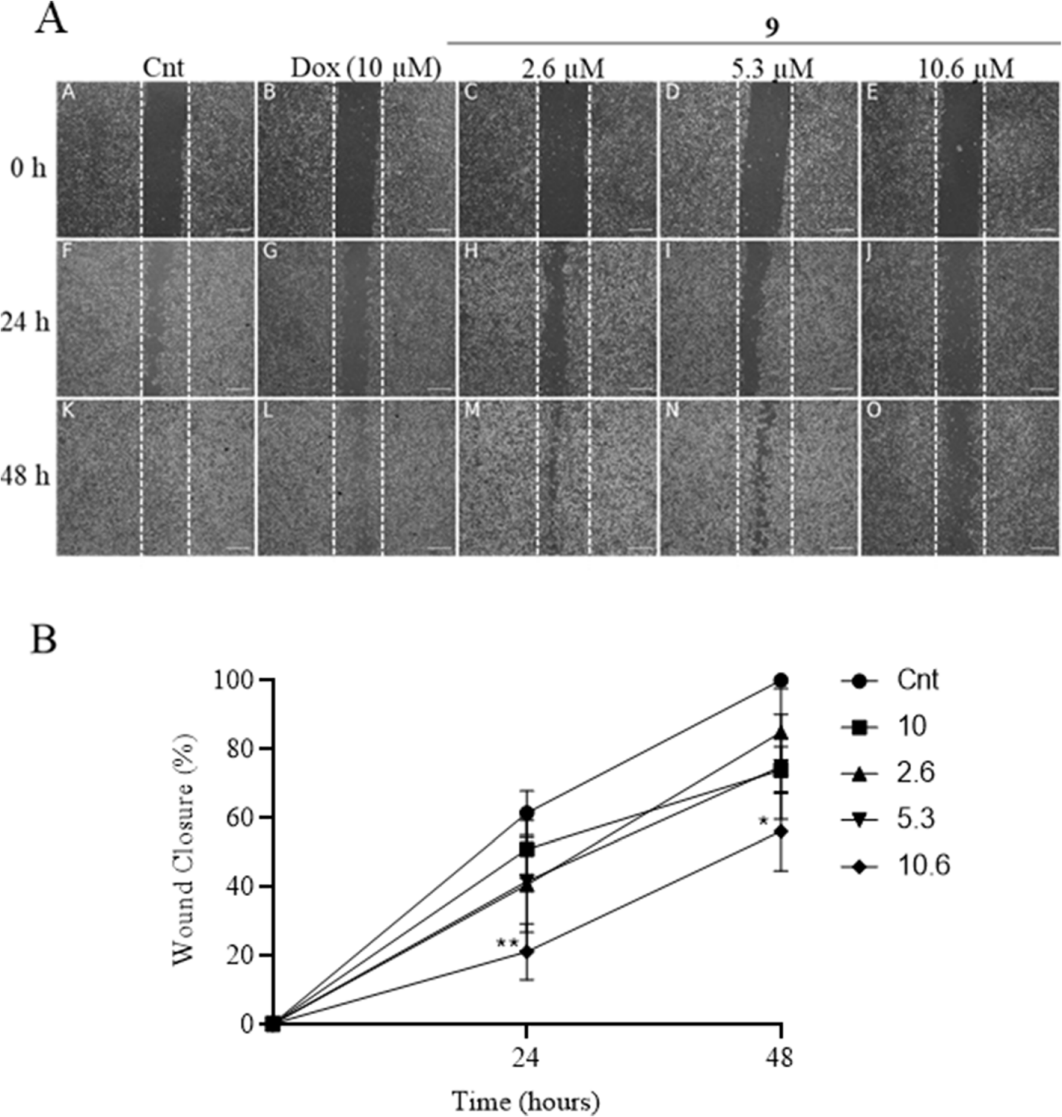
Inhibition of melanoma cells after treatment with compound 9. **A** Representative experiment of the Wound Healing test at 0, 24 and 48 h of treatment with compound 9 (2.6, 5.3 and 10.3 μM). 40x magnification. 100 μm scale bar. **B** Percentage wound closure. Data represent mean ± SD of three independent experiments. Doxorubicin (10 μM) was used as positive control. Analysis between groups was performed with Anova Two-Way, followed by a Dunnett post-test. Comparisons were made between treatments and control of different times (*) *p* < 0.05, (**) *p* < 0.01

To evaluate the migration capacity of melanoma cells after treatment with compound 9, the wound healing assay was performed. Three treatment concentrations (2.3, 5.3 and 10.6 μM) were used, and the analyzes were performed at times 0, 24 and 48 h. As result, it was observed that the cells of the negative control (DMSO 0.02%) migrated to the center of the scratch and the percentage of closure at 48 h was 100% (Fig. 4A). In the treatment with compound 9, a reduction in the migratory capacity of melanoma cells was observed at 24 and 48 h compared to the control (Fig. 4B). The highest concentration evaluated (10.6 μM) inhibited the closure of the scratch about 80% over 24 h compared to the control treatment free. Comparatively, compound 9 reduced wound closure more than the positive control doxorubicin. No significant reduction was observed in the lowest treatment concentrations (2.3 and 5.3 μM) with the compound 9.

Although no statistically significant reduction has been observed in the two lowest concentrations treatment (2.3 and 5.3 μM), it is visually evident in the Fig. 4A that the wound was not completely closed 48 h after the treatment.

### The cells treated with compound 9 show apoptosis characteristics

The main structural changes in the B16F10 cell lines after treatment with compound 9, were evaluated by staining the actin filaments and cell nucleus with Phalloidin/FITC and DAPI, respectively. The qualitative results of the treatment with 2.6, 5.3 and 10.6 μM have shown that the compound 9 promoted changes in the nucleus and cytoplasm of the cells.

**Fig. 5:**
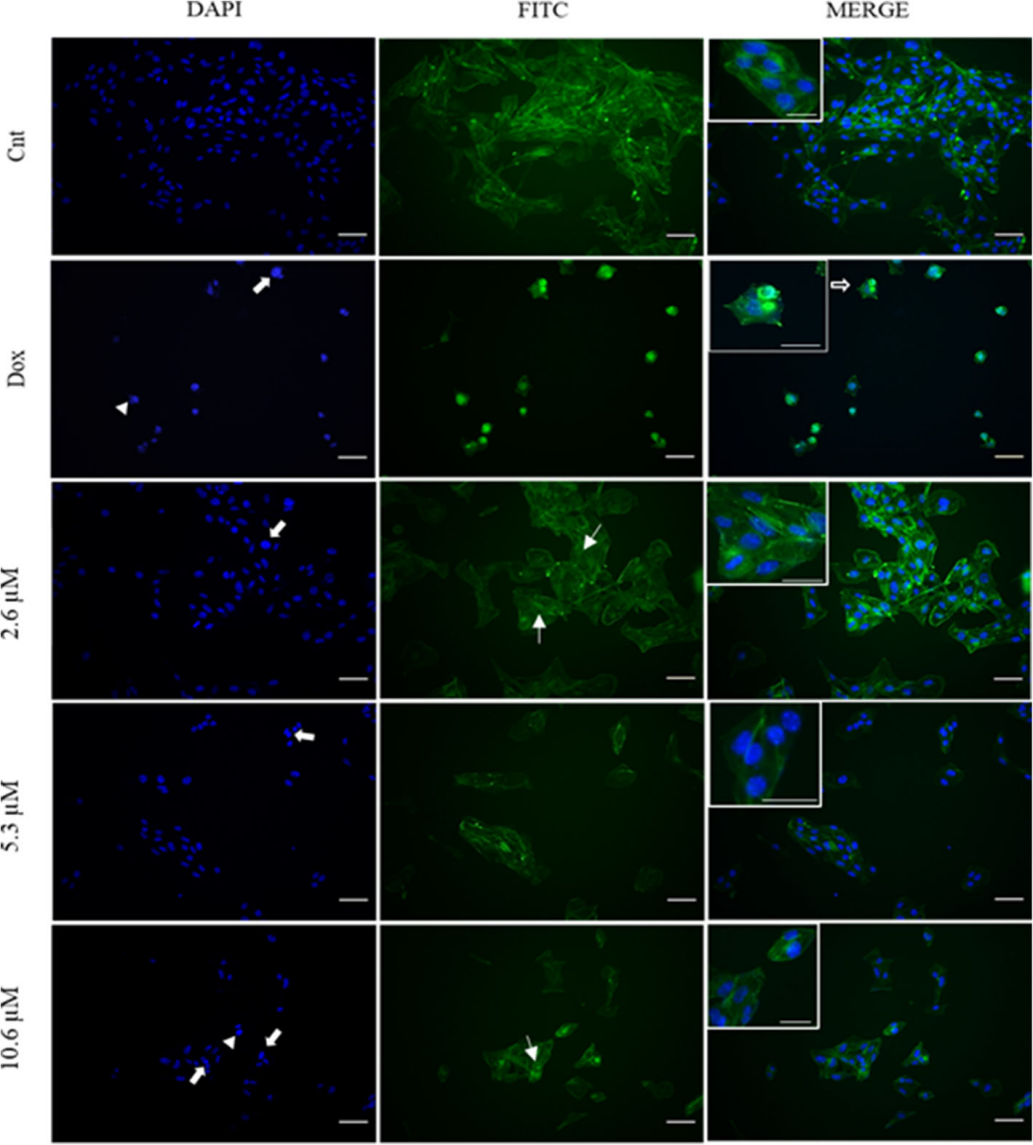
Effect of compound 9 on the cystoskeleton and cell nucleus morphology of the B16F10

It is possible to observe through the staining of the F-actin filaments that the organization of these fibers in melanoma cells became altered after treatment with the compound 9. Several stress bundles were observed in the cytoskeleton, which are arranged in a disorganized manner as they are exposed to the treatments with different concentrations of compound 9 (Fig. 5).

The nucleus of the cells showed changes characteristic of apoptosis, such as DNA fragmentation and chromatin condensation after treatment with compound 9 (Fig. 5). These changes were observed in all the concentrations; however, they were more frequent in the highest concentration of 10.6 μM. The drug doxorubicin (10 μM), used as positive control of apoptosis, caused changes in the cell nucleus that were similar to the higher concentration of the compound 9.

Morphological changes observed with DAPI and Phalloidin/FITC staining in B16F10 cells after treatment with compound 9 with the concentrations of 2.6, 5.3 and 10.6 μM after 24 h. Full arrows represent DNA fragmentation; arrowheads, chromatin condensation; hollow arrow apoptotic projections of cytoplasm and thin arrow, stress bundles. 200x magnification. Scale bar = 20 μm. Insert scale bar = 10 μm.

### The compound 9 increases early apoptosis in melanoma after treatment

To evaluate the type of cell death induced by the compound 9 in B16F10, a flow cytometry assay was performed. The melanoma cells were treated with three concentrations, 2.6, 5.3 and 10.6 μM, of the test compound, for 24 h. Then, they were stained with Annexin V/FITC and Propidium Iodide (PI).

**Fig. 6:**
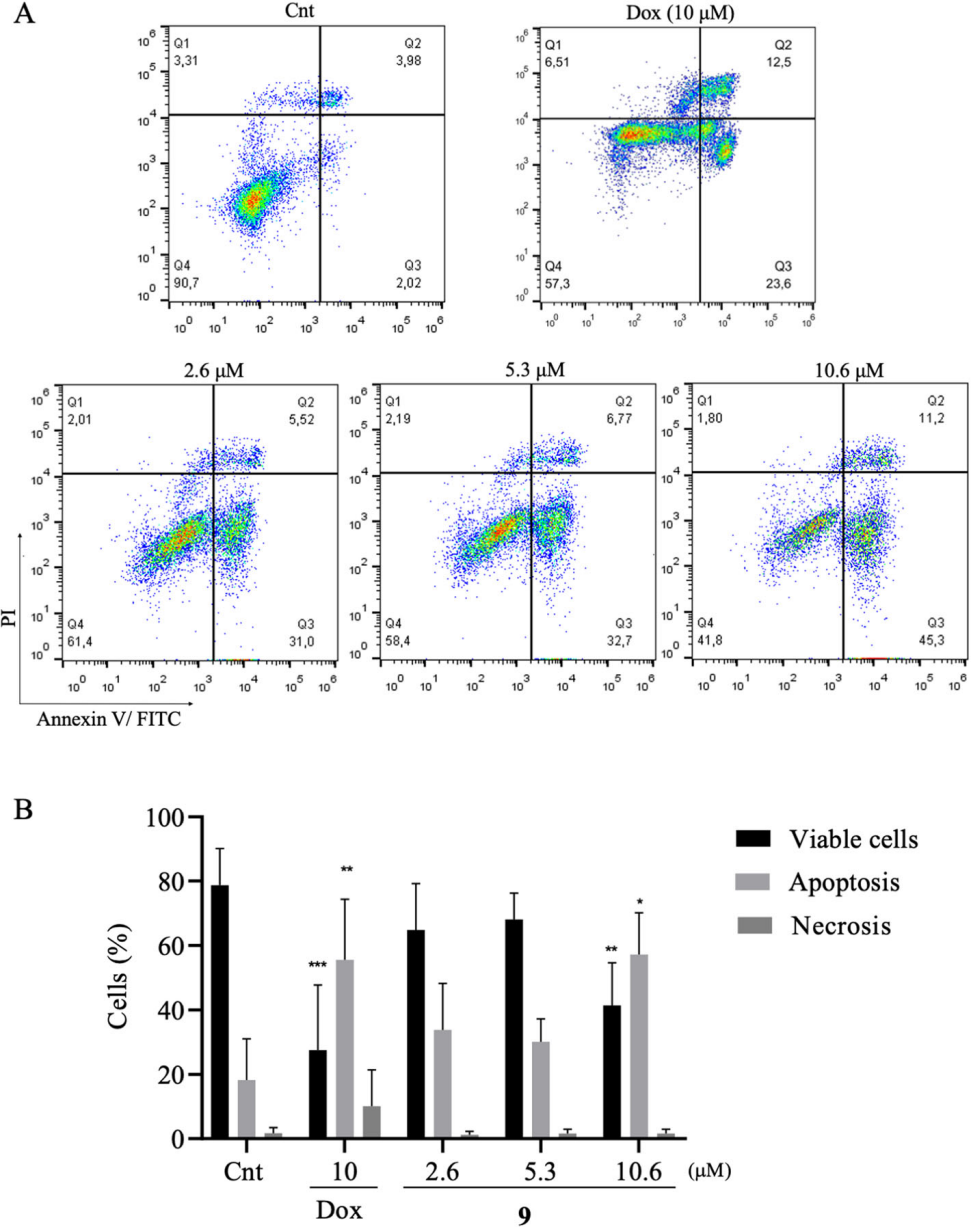
Evaluation of apoptosis and necrosis induction of compound 9 in melanoma cells. **A** Analysis of B16F10 cells by flow cytometry after treatment with compound 9 with the concentrations of 2.6, 5.3 and 10.6 μM for 24 h. Viable cells (Annexin V−/IP-), early apoptosis (Annexin V+/PI-), late apoptosis (Annexin V+/PI+) and necrosis (Annexin V−/PI+). The negative control was treated with vehicle of the tested compound (DMSO 0.05%). Doxorubicin (10 μM) was used as positive control. **B** Graphs of percentage of live, apoptosis and necrosis cell. Data presented with mean ± SD values from three independent experiments. The analysis between groups and negative control was performed with ANOVA, followed by Dunnett post-test. (*) *p* < 0.05, (**) *p* < 0.01 and (***) *p* < 0.001

Figure 6A illustrates a representative experiment of a dot plot graphs obtained in a flow cytometry experiment, indicating viable cells (Annexin V−/IP-), early apoptosis (Annexin V + / IP-), late apoptosis (Annexin V + / IP +) and necrosis (Annexin V- / IP-). It was possible to observe that compound 9 reduced the percentage of viable cells in the highest concentration (10.6 μM). In addition, the compound induced an increase in the percentage of melanoma cells dying by apoptosis (Fig. 6B). There was no increase in the percentage of cells in apoptosis at the other concentrations evaluated (2.6 and 5.3 μM). There was no increase in melanoma cells dying from necrosis at the tested concentrations. The drug doxorubicin (10 μM), used as positive control, reduced the percentage of viable melanoma cells and significantly increased the percentage of cells in necrosis (Annexin V−/IP-).

## Discussion

*Lawsonia inermis* (Henna) is an important plant species, source of bioactive compounds with therapeutic activity. As described by Ishteyaque et al., henna extract has showed cytotoxic activity against lung, colon and liver cancer cell lines and its activity can be attributed to lawsone, one of the active compounds in the plant responsible for the cytotoxic activity in tumor cells [32]. Studies with derivatives of this compound have been widely carried out in order to find potent compounds against different types of tumors [18]. The process of discovering a new drug goes through several stages ranging from the selection of the molecule and preclinical tests to more advanced tests in humans. Carrying out studies on the reuse of medications in another illness can reduce the time to discover potential medications [33]. In addition, studies of derivatives of known drugs may produce more potent and less toxic compounds [22].

It was possible to observe a reduction in the growth of lung carcinoma, melanoma and glioma cells after treatment with synthetic derivatives of lawsone. To evaluate the effect of the compounds, the SRB assay was used, an anionic dye used to evaluate cell viability based on the measurement of the content of proteins that bind to it electrostatically [24]. Among the sixteen compounds evaluated, the compounds 1, 9, 10, 11 and 15 showed growth inhibition higher than 90% (GI > 90%) in the three cell lines and IC_50_ were carried out for three of them (9, 10 and 11). The etherification of the hydroxyl group of lawsone led, in general, to compounds with enhanced activity (compounds 9–11 and 13–15) while lawsone and the derivatives bearing a free hydroxyl group at C-2 of the naphthoquinone ring (compounds 4–8) were less active. The presence of the C-2 hydroxyl group enhances the polarity of the compounds, which impair cell membrane permeation. The low activity of compounds 12 and 16, can be explained by their high polar character, since these compounds have deacetylated sugar moieties in their structures. This would impair cell membrane permeation. Ottoni and colleagues evaluated glycosidic lawsone derivatives, with chemical characteristics similar to those evaluated in this research, in different cell lines of breast adenocarcinoma (SKBR-3, MDA-MB-231, and MCF-7), they also observed greater cytotoxicity of compounds 9, 10 and 11 in tumor cells compared to non-tumor cells of human gingival fibroblasts (HCT) [22]. One important chemical characteristic of this compounds is that they are quinones and this structures has the capacity of release electrons and consequently form free radicals, such as superoxide anion and hydrogen peroxide, causing damage to cell structure [31, 34].

The melanoma, despite of its low incidence, is the most aggressive cancer among skin cancers and is responsible for about 80% of the causes of death [35]. In addition to being highly aggressive, malignant melanoma cancer is also resistant to drugs [36]. There are some characteristics in melanoma cells that make them more aggressive compared to other cell types of other cancers. This can be justified by some molecular characteristics that imply pathogenesis and clinical response to treatments [37]. Research for new compounds with cytotoxic potential may promote the discovery of new drugs for the treatment of melanoma.

The clonogenic assay showed that the compound 9 reduces the number and size of melanoma cell colonies in relation to the control, corroborating with the results found in the SRB assay. This result shows that the compound is capable of inhibiting the production of clones from a single cell, it also shows the cellular response to treatment in a longer evaluation period, in which the cells were maintained in a viable condition of growth [38]. Chipoline and colleagues [39] observed in their study with 1,4-naphthoquinone derivatives that naphthoquinone compounds are able to reduce the formation of oral squamous cell carcinoma colonies (SSC-9), without reducing the viability of non-cells fibroblast tumor cells. Caro and colleagues [40] also observed that the naphthoquinone derivative 8-hydroxy-2-(2-tenoyl)naphtho[2,3- *b*] thiophene-4,9-dione reduced the formation of human colorectal adenocarcinoma cell colonies (HT-29). This same compound did not reduce the viability of fibroblasts.

Melanoma cancer has a high capacity to generate metastases. This characteristic is mainly related to the migratory capacity and mobility that cells of this type of cancer have [41]. Among cancer-related deaths, about 90% are related to metastatic disease [42]. Several steps are necessary before the complete installation of melanoma cells in other tissues. These are linked to the complex behavioral dynamics of cells, mobility, cytoskeleton and connection to the tumor microenvironment, as well as production of growth factors and cytokines. Although metastasis is a key factor in the aggressiveness of melanoma, there are no drugs that specifically inhibit or reduce this migration and invasion of cells to other tissues [43, 44].

The results obtained in the wound healing assay showed that the compound 9 was able to inhibit the migration of melanoma cells (B16F10) at the highest concentration evaluated (10.3 μM). This activity can be attributed to decreased proliferative capacity by melanoma cells exposed to treatment, as well as by the activity of the compound on the disposition of the actin filaments that compound the cytoskeleton, as observed in the experiment with the Phalloidin/FITC probe. Since cytoskeleton is a fundamental part in cell movement and can influence the migratory capacity of cells [44].

Several studies demonstrate using different analyzes that naphthoquinone derivatives show cytotoxic activity in tumor cells through the induction of apoptosis [32, 45]. Chipoline and colleagues [39] observed that an 1,4-naphthoquinone derivative analyzed in their research induced apoptosis in SCC-9 cells (oral squamous cell carcinoma). The main changes observed were cell morphology alterations with disorganization of microtubules and inhibition of topoisomerase [39]. The topoisomerase enzyme is responsible for breaking and repairing DNA strands [46]. Im and colleagues [47] also showed apoptosis after treatment with a naphthoquinone-derived compound in human colon cancer cells (HCT116). This compound increased the production of reactive oxygen species (ROS), induced the activation of caspases 3, 8 and 9.

By staining the nucleus and filaments of F-actin, the cells showed apoptosis characteristics after treatment with different concentrations of the compound 9 and doxorubicin, used as a positive control. One of the most observed changes in the cell nucleus, in the concentrations tested, was DNA fragmentation. The fragmentation of genetic material is characteristic of the programmed cell death, since the apoptotic stimulus causes activation of the cascade of caspases that cleaves and activates the DNA fragmentation, allowing the breaking of DNA molecules into pieces with 180 pairs of bases [48]. In addition, another important characteristic of the apoptotic death is the high condensation of chromatin [49]. This was observed in the highest treatment concentration (10.6 μM) of the compound 9, as well as in the treatment with doxorubicin.

Alterations in the actin filaments are present in tumor cells and it implies the tumor invasion process [50]. Cellular shrinkage and morphological changes are characteristic of apoptosis [51]. The analysis of the cytoplasm of the B16F10 after treatment with the compound 9 revealed a reduction in the size and changes in the morphology of the cells, with loss of the fibroblast aspect. In addition, changes in the disposition of the actin filaments, important structures responsible for numerous functions in the cell, such as morphology and migration, were observed [52].

Apoptosis is a death mechanism observed in several tumor cell lines treated with naphthoquinonic compounds [14]. Oliveira and colleagues [53] observed that lawsone derivatives induce apoptosis in prostate cancer cells (DU-145), breast (MCF-7) and lung carcinoma (A549). The analyzes of cytotoxic activity of the compound in A549 showed that the compound was able to inhibit cell migration by 85% at the highest treatment concentration. In addition, it induced cell cycle arrest in the Sub G1 phase and increased the percentage of A549 cells in initial apoptosis by 50% at the highest treatment concentration with the lawsone derivative [53].

In the present study, the melanoma cell line (B16F10) showed high expression of Annexin V after treatment with the highest concentration of the compound 9, indicating an increase in cells in apoptosis. Annexin V is a member of the calcium-dependent Annexin protein superfamily [54]. This protein plays a role in binding to phosphatidylserine, externalized in the membrane of cells undergoing apoptosis. When an apoptotic stimulus occurs, the cells begin to externalize phosphatidylserine, which is normally located in the inner plasma membrane. It works as a signal for phagocytes to carry out efferocytosis, removal of apoptotic cell bodies from the body [55].

In conclusion, the results of this research show that derivatives of lawsone are an important source of possible compounds with cytotoxic activity in tumor cells. Compound 9, synthesized from the lawsone compound causes a reduction in cell proliferation and viability, mainly due to characteristic changes of apoptosis in melanoma cells (B16F10). This type of death can be less harmful because it is a largely regulated cell death, in which there is no leakage of the cytoplasmic and nuclear material of the cells, reducing or preventing an inflammatory process [56]. Although compound 9 has shown promising results, it is necessary to further evaluate the mechanisms of action of this compound with subsequent analysis in in vivo tumor models*.*

## Supplementary Information

**Additional file 1.**

## Data Availability

The datasets used and/or analysed during the current study available from the correspondingauthor on reasonable request.

## Abbreviations

A549: Human Lung Carcinoma Epithelial cell line
ANOVA: Analysis of variance
B16-F10: Melanoma cell line
C6: Glioma cell line
DAPI: 4′,6-Diamidine-2-Phenylindole Dihydrochloride
DMEM: Dulbecco’s Modified Eagle Medium
DMSO: Dimethylsulfoxide
DOX: Doxorubicin
EDTA: Ethylenediamine tetraacetic acid
FITC: Fluorescein Isothiocyanate
GI_50_: Degree of inhibition for 50% of cells
IC_50_: Inhibitory concentration for 50% of cells
PBS: Phosphate Buffered Saline
PI: Propidium Iodide
SFB: Fetal bovine serum
