# Supplementary material for: Anti-proliferative and pro-apoptotic activity of glycosidic derivatives of lawsone in melanoma cancer cell
Source: BMC Cancer. 2021 Jun 2;21:662. doi: 10.1186/s12885-021-08404-4 (PMC8173884; doi:10.1186/s12885-021-08404-4)
Supplement: Supplementary file 1 — Additional file 1. [file 12885_2021_8404_MOESM1_ESM.pdf]

Supporting Information

Anti-proliferative and pro-apoptotic activity of glycosidic derivatives of lawsone in melanoma cancer cell

Mariana Nobre Farias de Franca[1], Raquel Geralda Isidório[3], João Henrique Oliveira Bonifacio, Edmilson Willian Propheta dos Santos[1], Jileno Ferreira Santos[2], Flaviano Melo Ottoni[3], Waldecy de Lucca Junior[4], Ricardo Scher[2], Ricardo José Alves[3], Cristiane Bani Corrêa[1*]

[1]Graduate program in health sciences Federal University of Sergipe, Laboratory of Biology and Immunology of Cancer and Leishmania, Departamet of Morphology, Federal University of Sergipe

[2]Laboratory of Biology and Immunology of Cancer and Leishmania, Department of Morphology, Federal University of Sergipe

[3]Laboratory of Pharmaceutical Chemistry, Department of Pharmaceutical Products, Faculty of Pharmacy, Federal University of Minas Gerais

[4]Laboratory of Molecular Neuroscience of Sergipe, Department of Morphology, Federal University of Sergipe

Contents:

1. Experimental section 2-5

Infrared data, ^{1}H NMR and ^{13}C NMR data for

2. Compound **2** 6-9
3. Compound **3** 10-13
4. Compound **5** 14-19
5. Compound **6** 20-22
6. Compound **7** 23-26
7. Compound **8** 27-30

Experimental section

Experimental Melting points were determined using Gehaka PF-1500 FARMA. The specific rotation ([α]) were determined using ADP 220 Polarimeter. IR spectra were recorded using a FTIR Spectrum One Perkin Elmer-ATR. ^{1}H and ^{13}C NMR spectra were measured on a Bruker AVANCE DRX400 NMR spectrometer or Bruker AVANCE DRX200 NMR spectrometer in $CDCl_3$ or DMSO-d_6 at 25°C. Chemical shifts (in ppm, δ scale) were referenced to the residual signals for $CHCl_3$ or DMSO. Coupling constants (J) are given in Hz. All ^{13}C NMR signals are singlets.

General procedure for the synthesis of lawsone triazoles

To a 50 mL round bottom flask was added the appropriate propargyl lawsone derivative (**1** or **4**) (0,05 g, 0.24 mmol) dissolved in 1 mL of tetrahydrofuran, followed by the appropriate azide (0.22 mmol), dissolved in 0.5 mL of tetrahydrofuran. Then, $CuSO_4.5H_2O$ (0.11 mmol), dissolved in 0.5 mL of water and sodium ascorbate (0.13 mmol), dissolved in 1 mL of water were added in a stepwise manner. The reaction mixture was stirred at room temperature for 4 h and monitoring by TLC analysis. The tetrahydrofuran was removed by distillation at reduced pressure. For peracetylated glycosyl triazoles the reaction residues were solubilized in 50 mL CH_2Cl_2 and washed with 2 x 50 mL H_2O and subsequently washed with 3 x 50 mL EDTA 20% w/v. The organic phase was dried over Na_2SO_4 and filtered. The organic phase was removed by distillation at reduced pressure. The peracetylated derivatives were added in Florisil and purified with silica column with following mobile phase (hexane:ethyl acetate, 7:3).

Synthesis of 2-[(1-(4-chlorophenyl)-1,2,3-triazol-4-methyl)oxy]-1,4-naphtoquinone, ***2***

Yield: 77% yellow solid; **mp**: 191 °C degrade without melting point; **IR (ATR) ν/cm^{-1}** 3355, 3104, 2924, 1676, 1657, 1603, 1593, 1578, 1501, 1405, 1363, 1329, 1302, 1246, 1202, 1159, 1095, 1040, 1011, 990, 927, 858, 828, 779, 749, 738, 719; **^{1}H NMR (400 MHz, DMSO-d_6) δ**

ppm: 9,04 (s, 1H, Triazole-H), 7,98 (m, 4H, 2NFq-H, 2Ar-H), 7,85 (t, 2H, *J* 7, 2-NFq-H), 7,69 (d, 2H, *J* 8, 2Ar-H), 6,66 (s, 1H, NFq-H), 5,35 (s, 2H, CH_2).**^{13}C NMR (100 MHz, DMSO-d$_6$) *δ* ppm:** 184,5, 179,4, 158,8, 142,1, 135,3, 133,7, 133,2, 131,5, 130,8, 129,9, 126,1, 125,6, 123,8, 122,0, 111,0, 62,15.

*Synthesis of 2-[(1-(phenyl)-1,2,3-triazol-4-methyl)oxy]-1,4-naphtoquinone, **3***

Yield: yellow solid (0,10 mmol, 43 %); **mp**: 120°C degrade without melting point; **IR (ATR) ν/cm^{-1}** 3280, 3136, 3091, 1650, 1644, 1603, 1593, 1576, 1505, 1468, 1376, 1358, 1332, 1308, 1258, 1230, 1201, 1190, 1177, 1154, 1124, 1085, 1052, 1039, 1034, 1009, 911, 902, 865, 842, 821, 810, 783, 757, 723, 703, 686; **^{1}H NMR (400 MHz, DMSO-d$_6$) *δ* ppm:** 9,01 (s, 1H, Triazole-H), 7,99 (t, 2H, *J* 7, 2NFq-H), 7,92 (d, 2H, *J* 7, 2Ar-H), 7,84 (m, 2H, 2NFq-H), 7,61 (t, 2H, *J* 7, 2Ar-H), 7,51 (t, 1H, *J* 7, Ar-H), 6,66 (s, 1H, NFq-H), 5,36 (s, 2H, CH_2). **^{13}C NMR (100 MHz, DMSO-d$_6$) *δ* ppm:** 185,2, 180,1, 159,6, 142,7, 137,2, 135,2, 134,3, 132,2, 131,5, 130,6, 129,6, 126,8, 126,4, 124,4, 120,9, 111,7, 62,9.

*2-hydroxy-3-[1-(2,3,4,6-tetra-O-acetyl-β-D-glucopyranosyl)-1,2,3-triazol-4-(methyl)]-1,4-naphtoquinone, **5***

Yield: yellow solid (0,18 mmol, 78 %); **[α]$_D^{25}$** –3.07 (C=0,26, $CHCl_3$); **mp**: 217,0 - 217,6 °C; **IR (ATR) ν/cm^{-1}**: 3351, 3070, 2921, 2851, 1742, 1646, 1589, 1515, 1460, 1424, 1372, 1350, 1319, 1255, 1235, 1215, 1162, 1134, 1094, 1075, 1060, 1037, 1023, 963, 951, 929, 908, 894, 815, 799, 765, 731, 687, 670; **^{1}H NMR (200 MHz, DMSO-d$_6$) *δ* ppm:** 11,3 (s, 1H, O-H), 8,06 (s, 1H, Triazole-H), 7,97 (d, 2H, *J* 7, 2Ar-H), 7,82 (d, 1H, *J* 7, 2Ar-H), 6,23 (d, 1H, *J* 9, Glic-H), 5,58 (t, 1H, *J* 9, Glic-H), 5,49 (t, 1H, *J* 9, Glic-H) 5,11 (t, 1H, *J* 9, Glic-H), 4,32 (m, 1H, Glic-H), 4,07 (m, 2H, Glic-H), 3,83 (s, 2H, CH_2), 2,00 (s, 3H, $OCOCH_3$), 1,96 (s, 3H, $OCOCH_3$), 1,93 (s, 3H, $OCOCH_3$), 1,74 (s, 3H, $OCOCH_3$). **^{13}C NMR (50 MHz, DMSO-d$_6$) *δ***

ppm: 184,0, 181,0, 170,0, 169,5, 169,3, 168,3, 156,1, 145,1, 134,6, 133,2, 131,9, 130,1, 125,8, 125,8, 121,5, 120,2, 83,6, 73,2, 72,2, 70,0, 67,6, 61,8, 20,5, 20,3, 20,2, 19,8, 19,4.

*2-hydroxy-3-[1-(2,3,4,6-tetra-O-acetyl-β-*D*-galactopyranosyl)-1,2,3-triazol-4-(methyl)]-1,4-naphtoquinone,* ***6***

Yield: yellow solid (0,14 mmol, 61 %); **$[\alpha]_D^{25}$** –1,6 (C=0,5, CH_2Cl_2); **mp**: degrades without melting in 198°C; **IR (ATR) ν/cm^{-1}** : 3348, 2989, 1744, 1644, 1590, 1538, 1459, 1426, 1371, 1346, 1309, 1274, 1213, 1162, 1098, 1046, 1016, 948, 919, 900, 851, 814, 798, 762, 728, 687, 669, 657; **^{1}H NMR (400 MHz, $CDCl_3$) *δ* ppm:** 8,09 (d, 1H, *J* 7, Ar-H), 8,02 (d, 1H, *J* 7, Ar-H), 7,76 (s, 1H, Triazole-H), 7,72 (d, 1H, *J* 7, Ar-H), 7,65 (d, 1H, *J* 7, Ar-H), 5,85 (d, 1H, *J* 9, Glic-H), 5,57 (m, 2H, *J* 9, 2Glic-H), 5,26 (d, 1H, *J* 9, Glic-H), 4,18 (m, 2H, 2Glic-H), 4,07 (s, 2H, CH_2), 2,23 (s, 3H, $OCOCH_3$), 2,02 (s, 3H, $OCOCH_3$), 1,99 (s, 3H, $OCOCH_3$), 1,84 (s, 3H, $OCOCH_3$). **^{13}C NMR (100 MHz, $CDCl_3$) *δ* ppm:** 184,0, 181,3, 170,2, 170,0, 169,7, 168,9, 154,1, 145,2, 134,8, 132,9, 132,5, 129,5, 126,6, 126,1, 120,5, 120,3, 86,1, 73,9, 70,8, 67,7, 66,9, 61,1, 20,6, 20,5, 20,4, 20,1, 19,7.

*2-hydroxy-3-[1-(2-acetamido-3,4,6-tri-O-acetyl-2-deoxy-β-*D*-glucopyranosyl)-1,2,3-triazol-4-(methyl)]-1,4- naphtoquinone,* ***7***

Yield: Yellow solid (0,05 mmol, 36 %); **$[\alpha]_D^{25}$** +2,0 (C=0,3 CH_2Cl_2); **mp**: degrades without melting at 100 °C; **IR (ATR) ν/cm^{-1} :** 3343, 3109, 3070, 2998, 2935, 1750, 1660, 1642, 1590, 1557, 1458, 1426, 1370, 1349, 1314, 1299, 1267, 1245, 1211, 1170, 1122, 1087, 1054, 1043, 1022, 1011, 989, 970, 948, 930, 918, 900, 865, 840, 814, 796, 763, 724, 684, 671, 659; **^{1}H NMR (400 MHz, DMSO-d_6) *δ* ppm:** 8,03 (d, 1H, *J* 6,96, Ar-H), 7,97 (d, 1H, *J* 7, Ar-H), 7,91 (s, 1H, Triazole-H), 7,82 (m, 2H, 2Ar-H), 6,05 (d, 1H, *J* 9, Glic-H), 5,32 (t, 1H, *J* 9, Glic-H), 5,03 (t, 1H, *J* 9, Glic-H), 4,46 (q, 1H, Glic-H), 4,18 – 4,04 (m, 3H, 3Glic-H), 3,83 (s, 2H, CH_2),

1,99 (s, 3H, $OCOCH_3$), 1,97 (s, 3H, NHCOC**H**$_3$), 1,91 (s, 3H, $OCOCH_3$), 1,53 (s, 3H, $OCOCH_3$); **^{13}C NMR (100 MHz, DMSO-d$_6$) *δ* ppm:** 183,9, 181,0, 170,2, 169,4, 168,4, 156,1, 144,8, 134,5, 133,2, 131,9, 130,1, 125,8, 125,7, 121,6, 120,2, 84,3, 71,3, 70,9, 69,9, 67,6, 20,4, 20,3, 19,9, 19,4, 15,7.

*2-hydroxy-3-[[1-(2,3,4-tri-O-acetyl-6-deoxy-β-*L*-galactopyranosyl)-1,2,3-triazol-4-yl]methyl]-1,4- naphtoquinone, **8**:*

Yield: Yellow solid (0.038 mmol, 24.12 %); **[α]$_D^{25}$** -6,9 (C=0,26 CH_2Cl_2); **mp**: degrades without melting at 266 °C; **IR (ATR) ν/cm^{-1}** 3349, 3069, 2881, 1816, 1741, 1665, 1646, 1589, 1524, 1459, 1425, 1372, 1350, 1274, 1231, 1217, 1163, 1103, 1046, 1023, 965, 951, 929, 905, 816, 799, 764, 731, 688, 672; **^{1}H NMR (400 MHz, DMSO-d$_6$) *δ* ppm:** 8,02 (dd, 1H, *J* 7,2 *J* 1 Ar-H), 7,97 (d, 1H, *J* 7,2, Ar-H), 7,91 (s, 1H, Triazole-H), 7,84 (td, 1H, *J* 7,2, *J* 1, Ar-H), 7,79 (td, 1H, *J* 7,2, *J* 1, Ar-H), 6,06 (d, 1H, *J* 10, Glic-H), 5,52 (t, 1H, *J* 10, Glic-H), 5,36 (dd, 1H, *J* 10, *J* 3, Glic-H), 5,2 (d, 1H, *J* 3, Glic-H), 4,34 (q, 1H, *J* 6,4, Glic-H), 3,84 (s, 2H, CH_2), 2,1 (s, 3H, $OCOCH_3$), 1,91 (s, 3H, $OCOCH_3$), 1,77 (s, 3H, $OCOCH_3$), 1,08 (d, 1H, *J* 6,4, CH_3); **^{13}C NMR (100 MHz, DMSO-d$_6$) *δ* ppm:** 183,8, 181,0, 170,0, 169,4, 168,4, 156,1, 144,8, 134,5, 133,2, 131,9, 130,1, 125,8, 125,7, 121,6, 120,2, 84,3, 71,3, 70,9, 69,9, 67,6, 20,4, 20,3, 19,9, 19,4, 15,6.

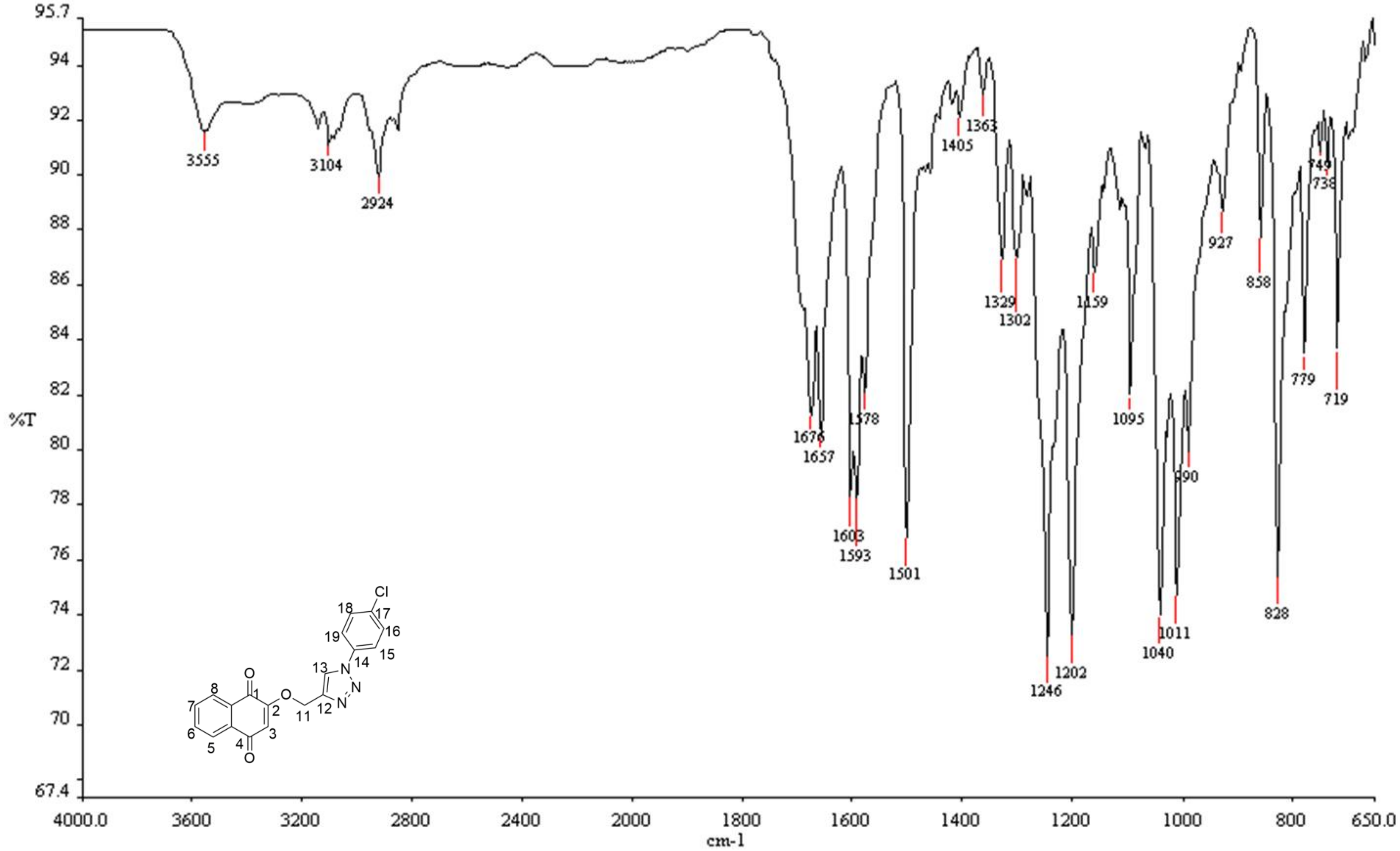

Infrared spectrum of 2

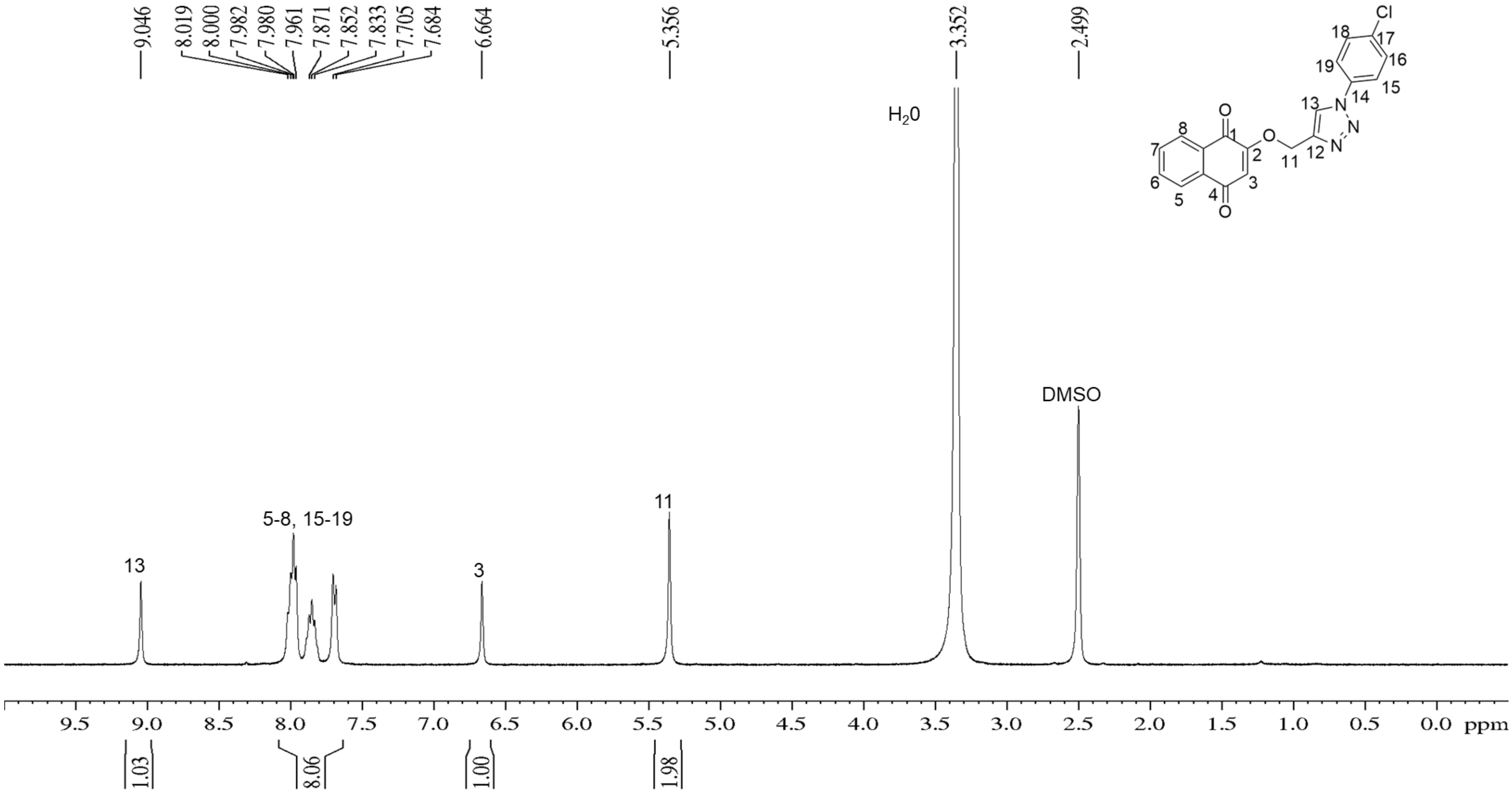

^{1}H NMR spectrum of 2 (400 MHz, DMSO-d6)

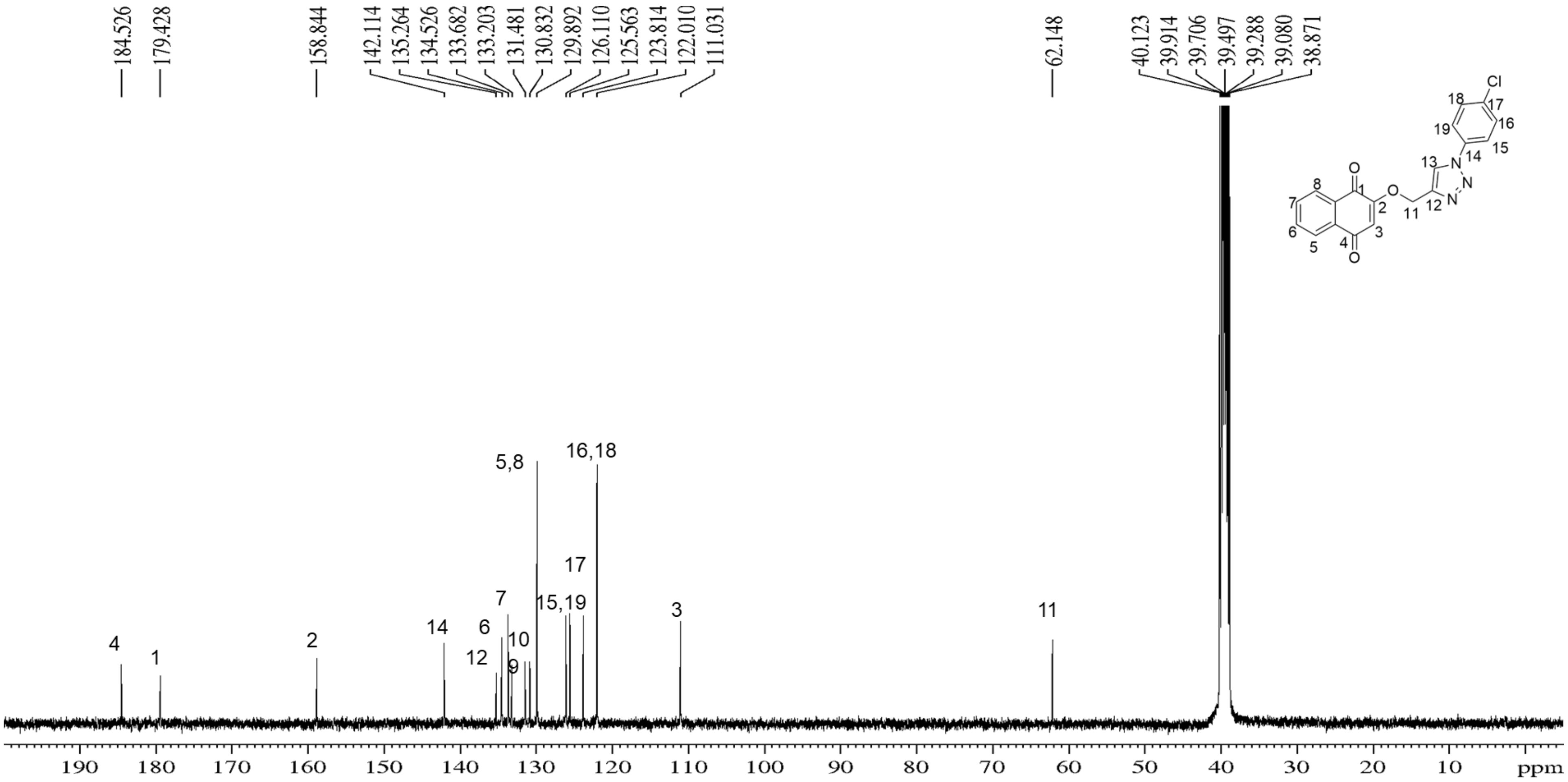

^{13}C NMR spectrum of 2 (100 MHz, DMSO-d$_6$)

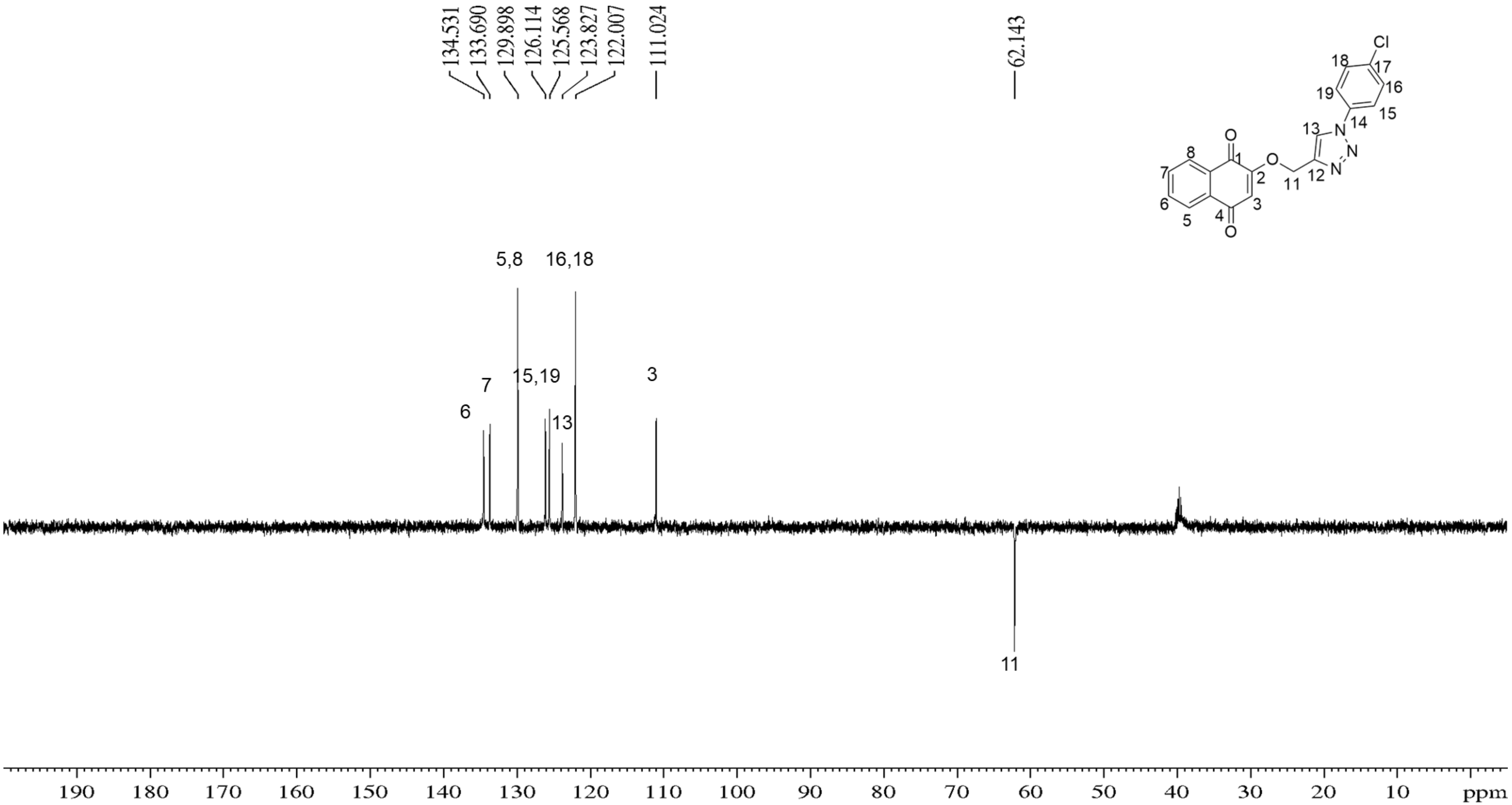

DEPT-135 of 2 (DMSO-d_6)

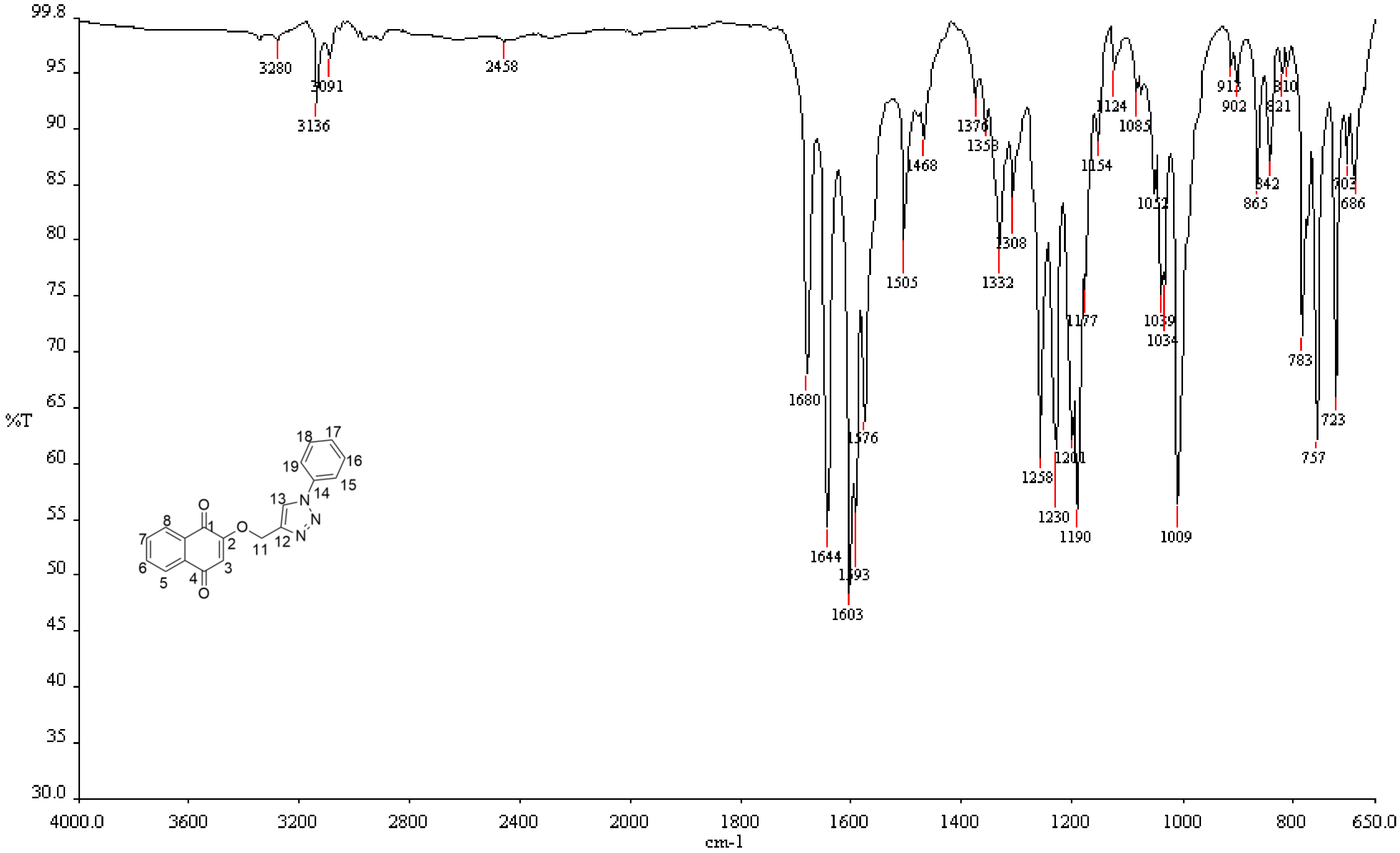

Infrared spectrum of 3

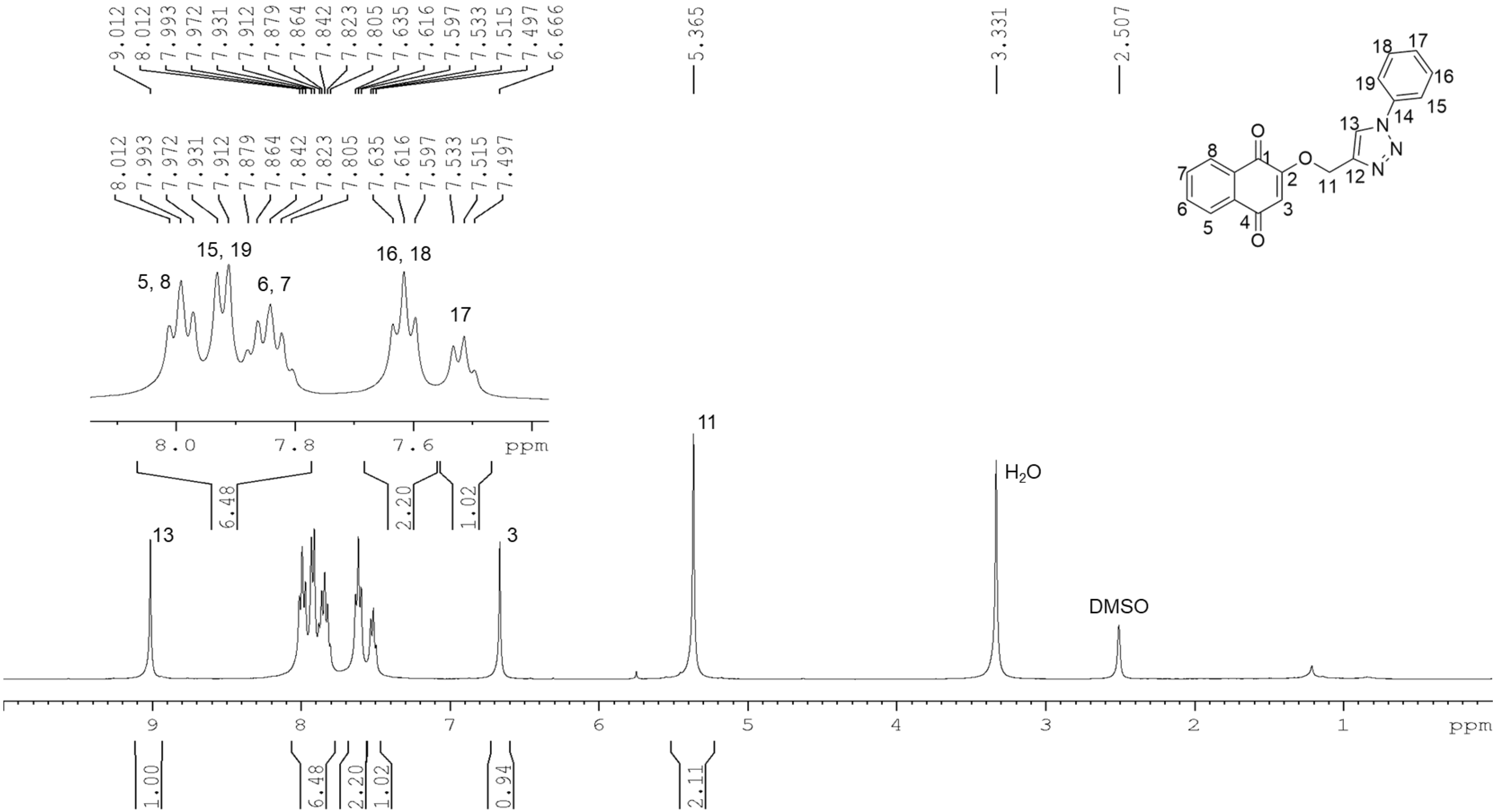

^{1}H NMR spectrum of 3 (400 MHz, DMSO-d6)

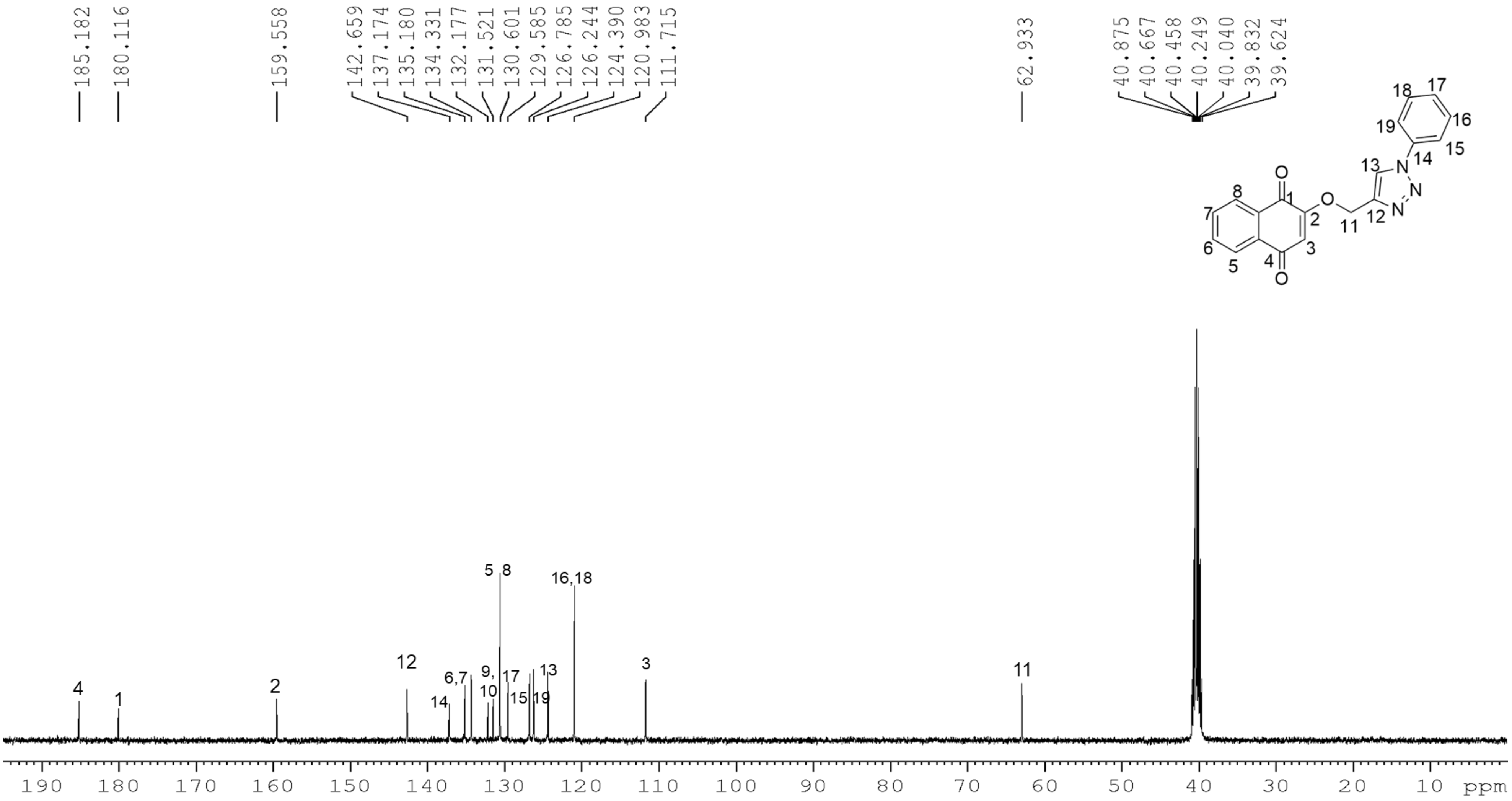

^{13}C NMR spectrum of 3 (100 MHz, DMSO-d_6)

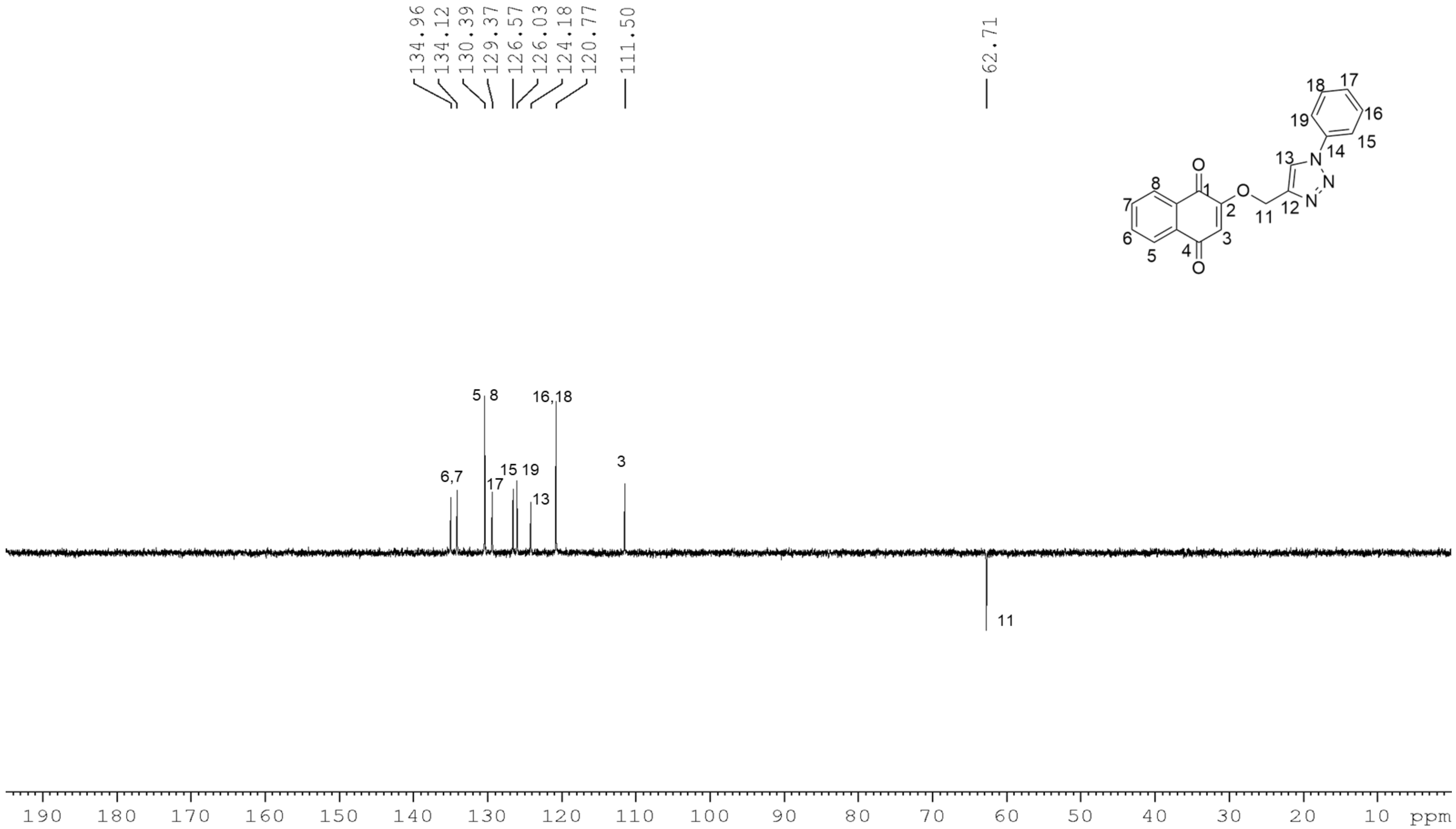

DEPT-135 of 3 (DMSO-d_6)

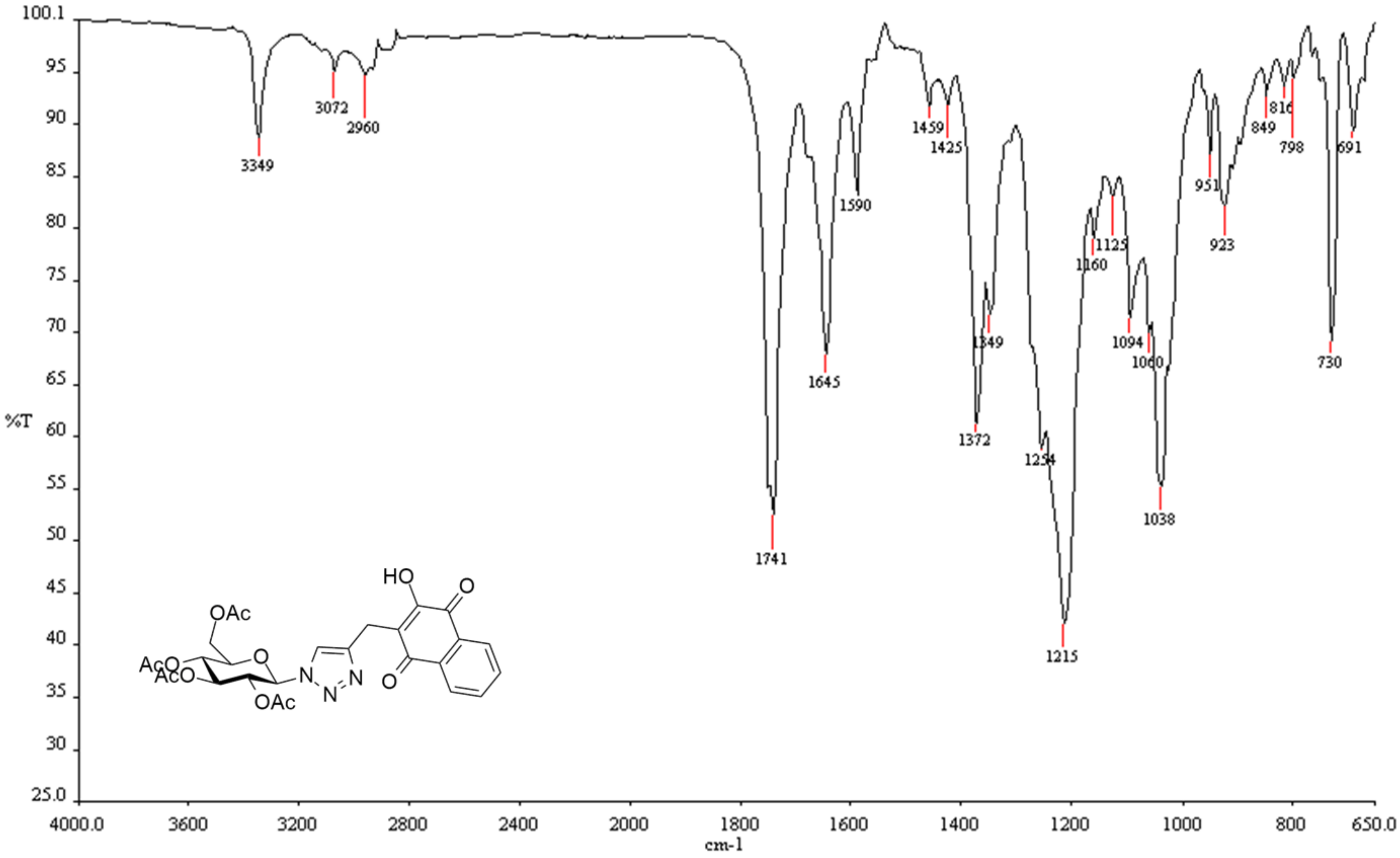

Infrared spectrum of 5

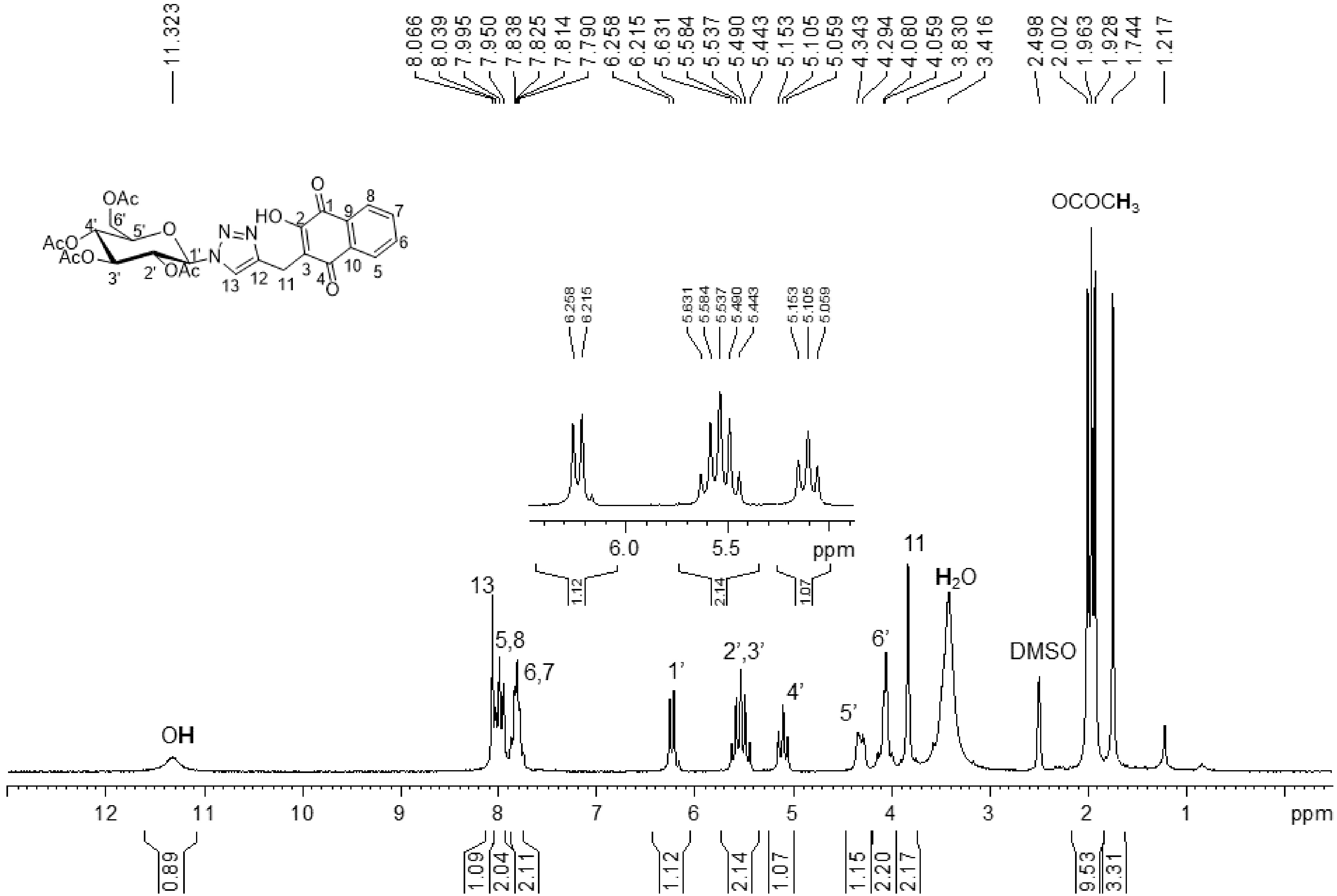

^{1}H NMR spectrum of 5 (200 MHz, DMSO-d$_6$)

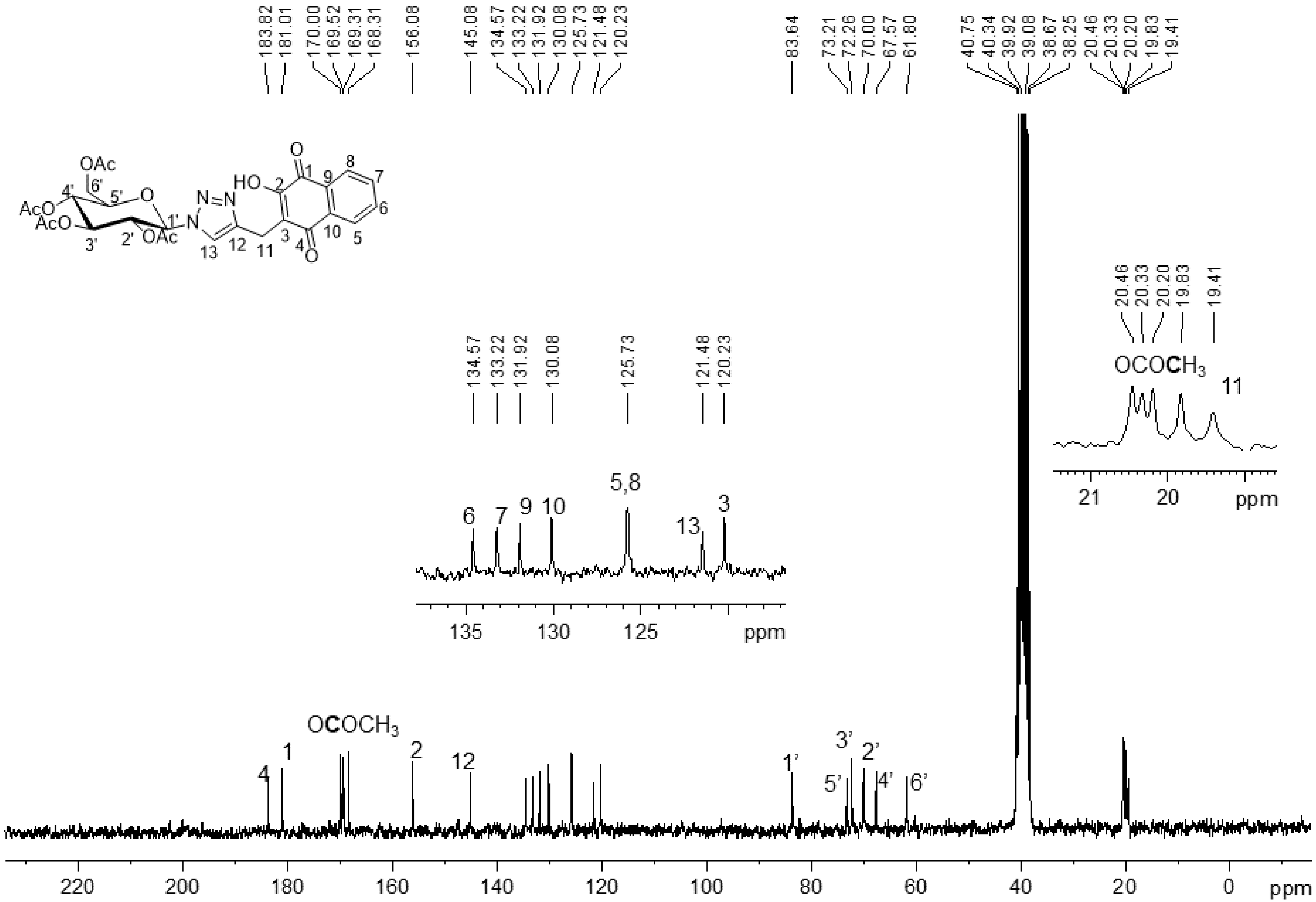

3C NMR spectrum of 5 (50 MHz, DMSO-d6)

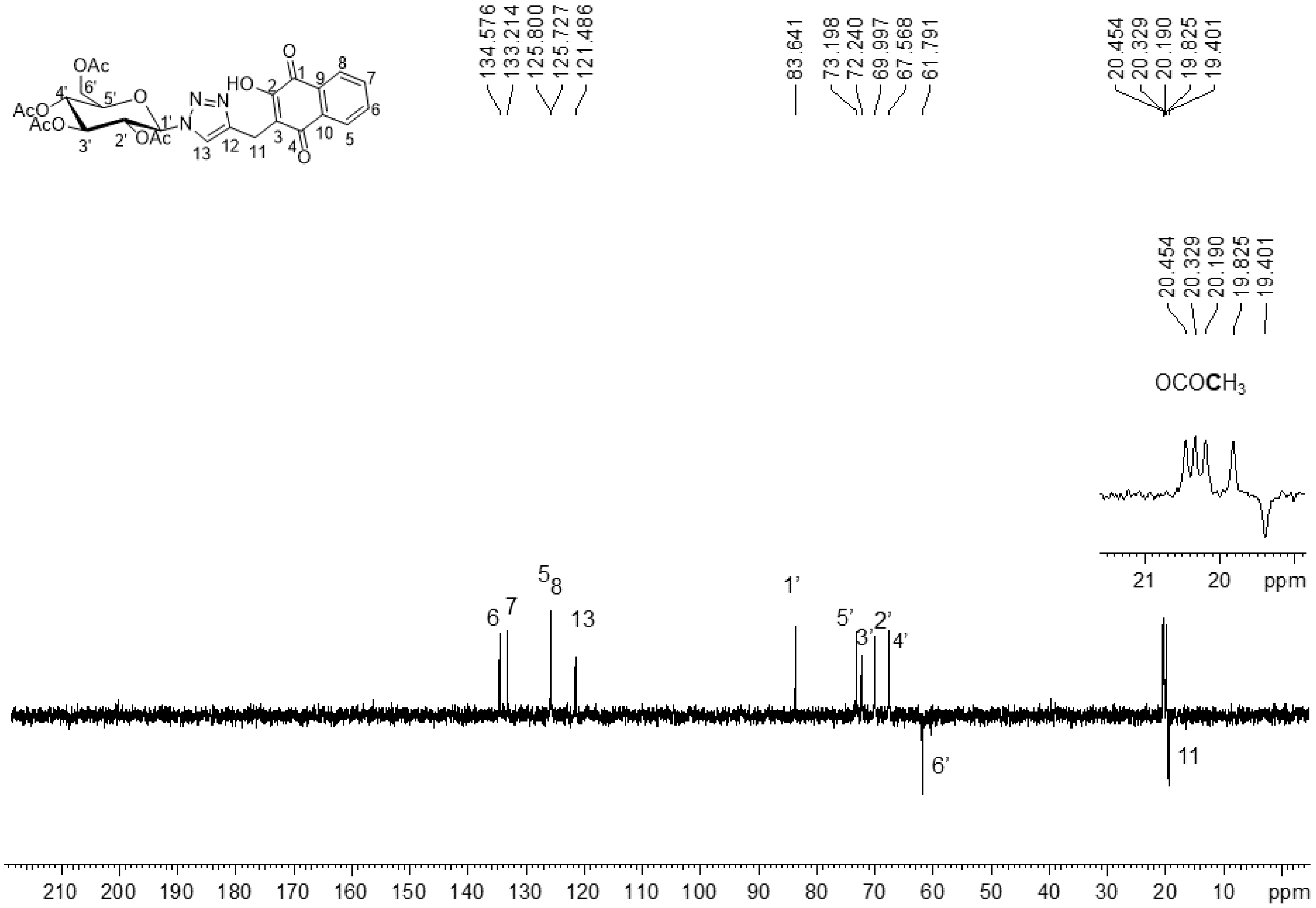

DEPT-135 of 5 (DMSO-d_6)

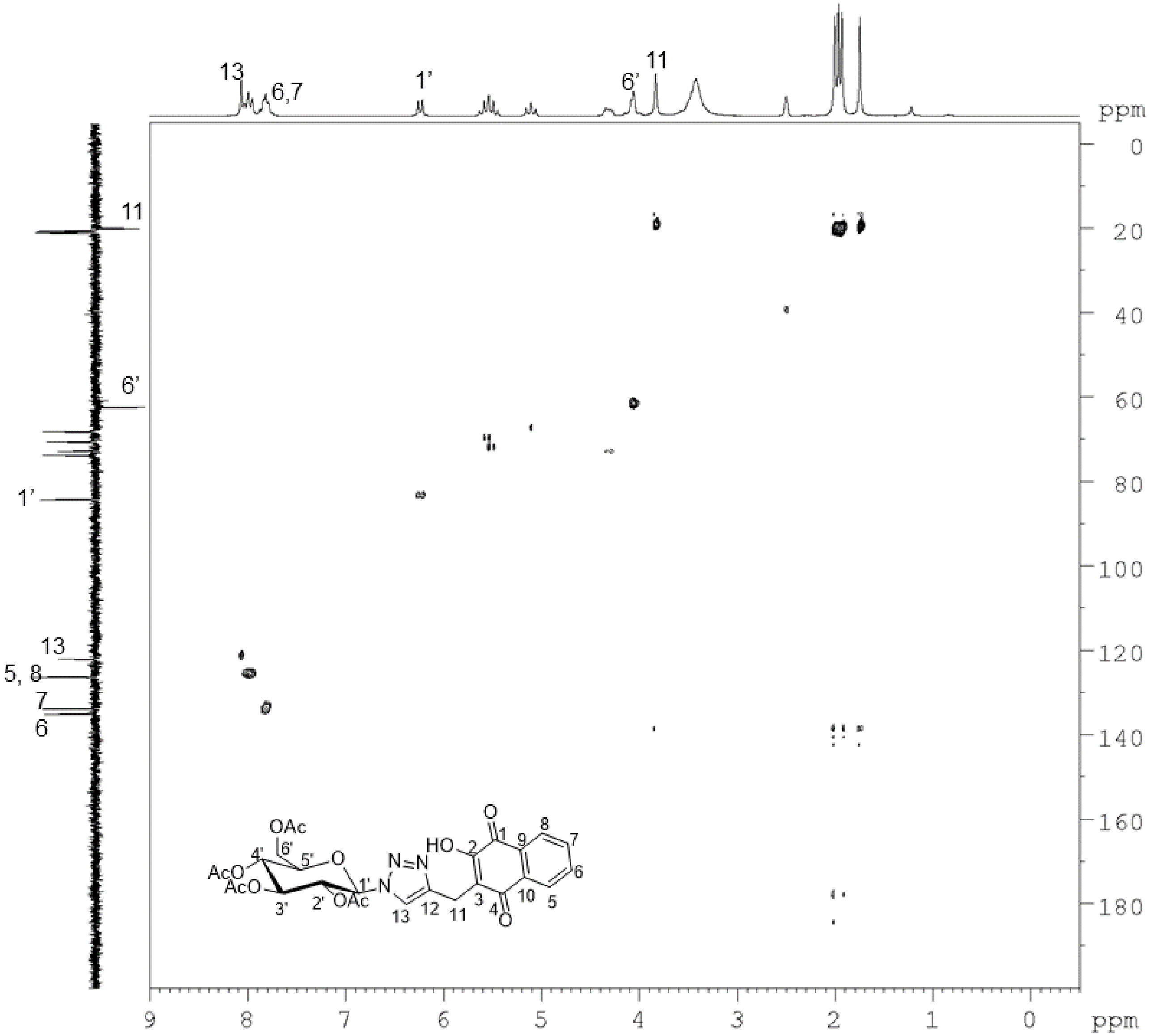

13
6,7
1'
6'
11
ppm
11
6'
1'
13
5, 8
7
6
OAc
6'
4'
5'
AcO
AcO
3'
2'
OAc
1'
HO
N
N
N
13
12
11
O
2
1
3
4
O
8
9
7
6
10
5
0
20
40
60
80
100
120
140
160
180
9
8
7
6
5
4
3
2
1
0
ppm

HSQC of 5

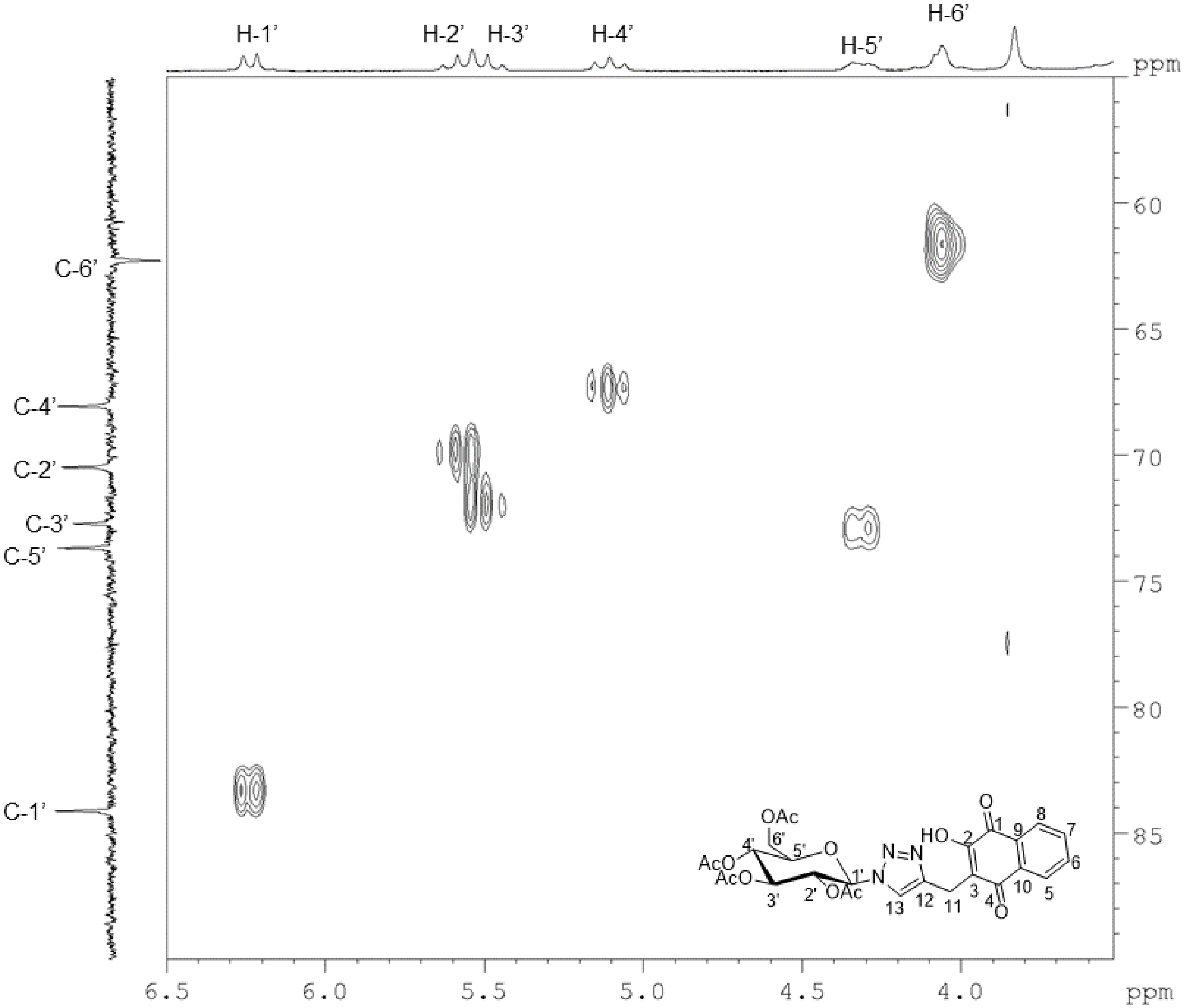

HSQC (3.5 at 6.5 ppm) of 5.

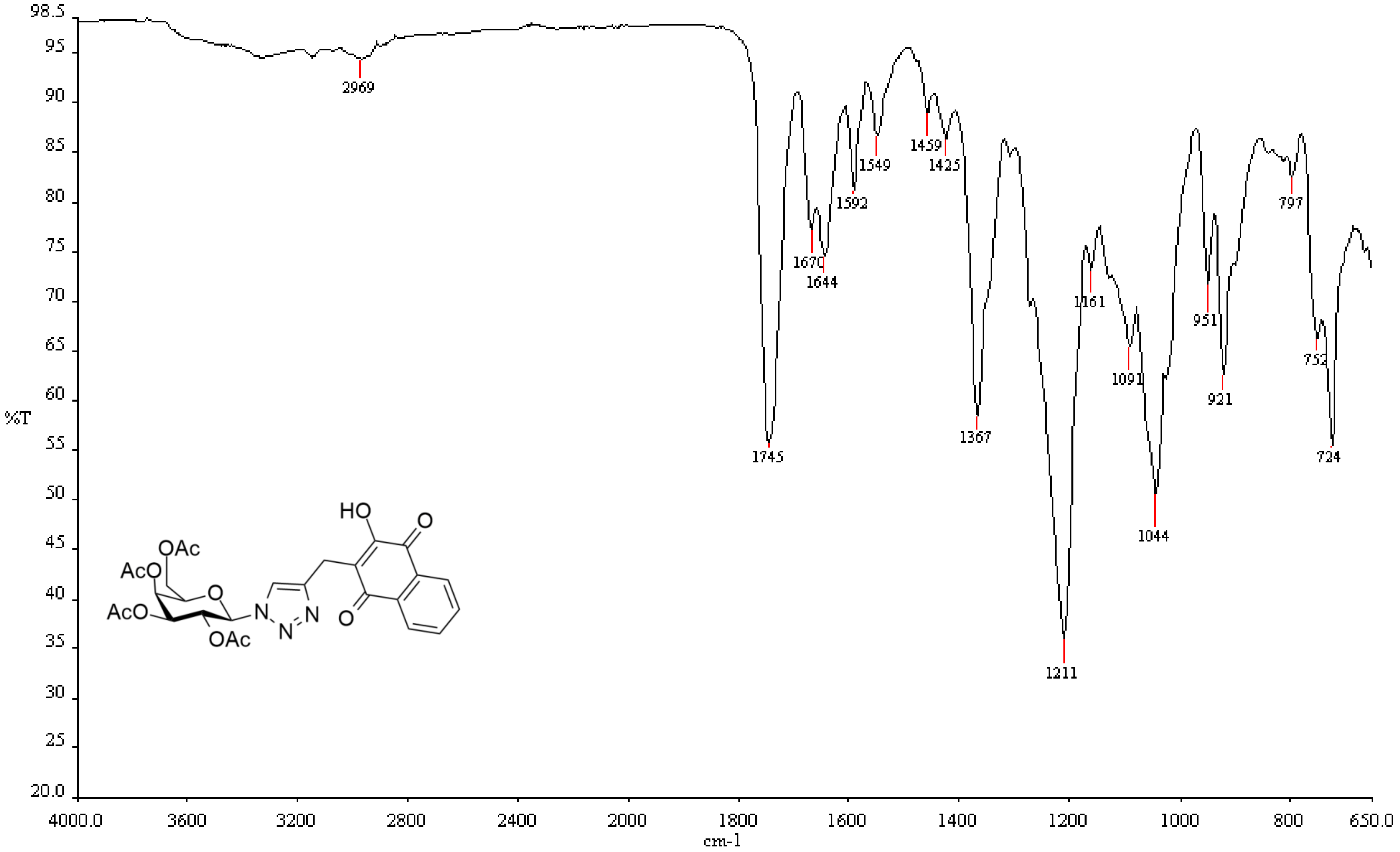

Infrared spectrum of 6

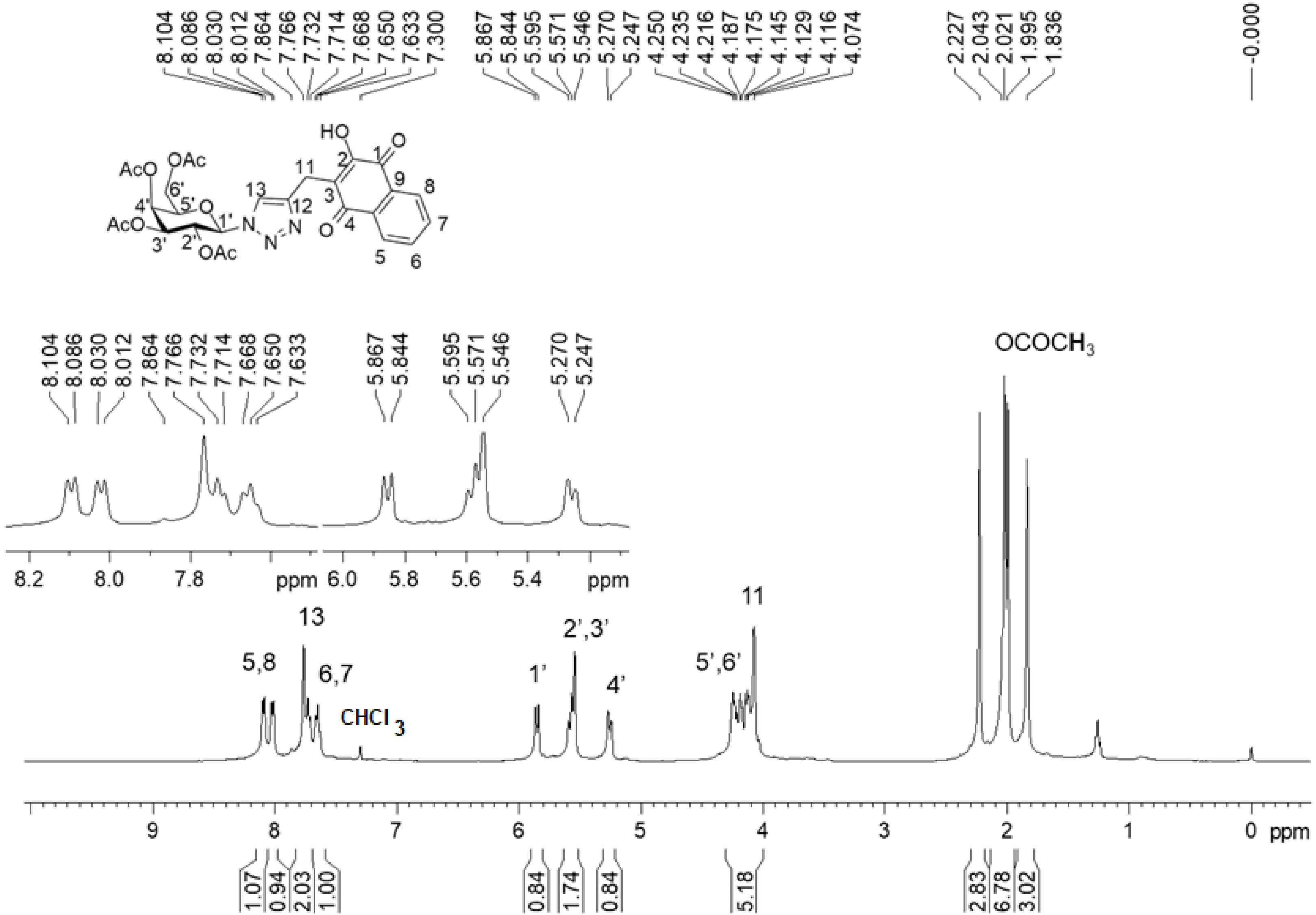

^{1}H NMR spectrum of 6 (400 MHz, CDCl$_3$)

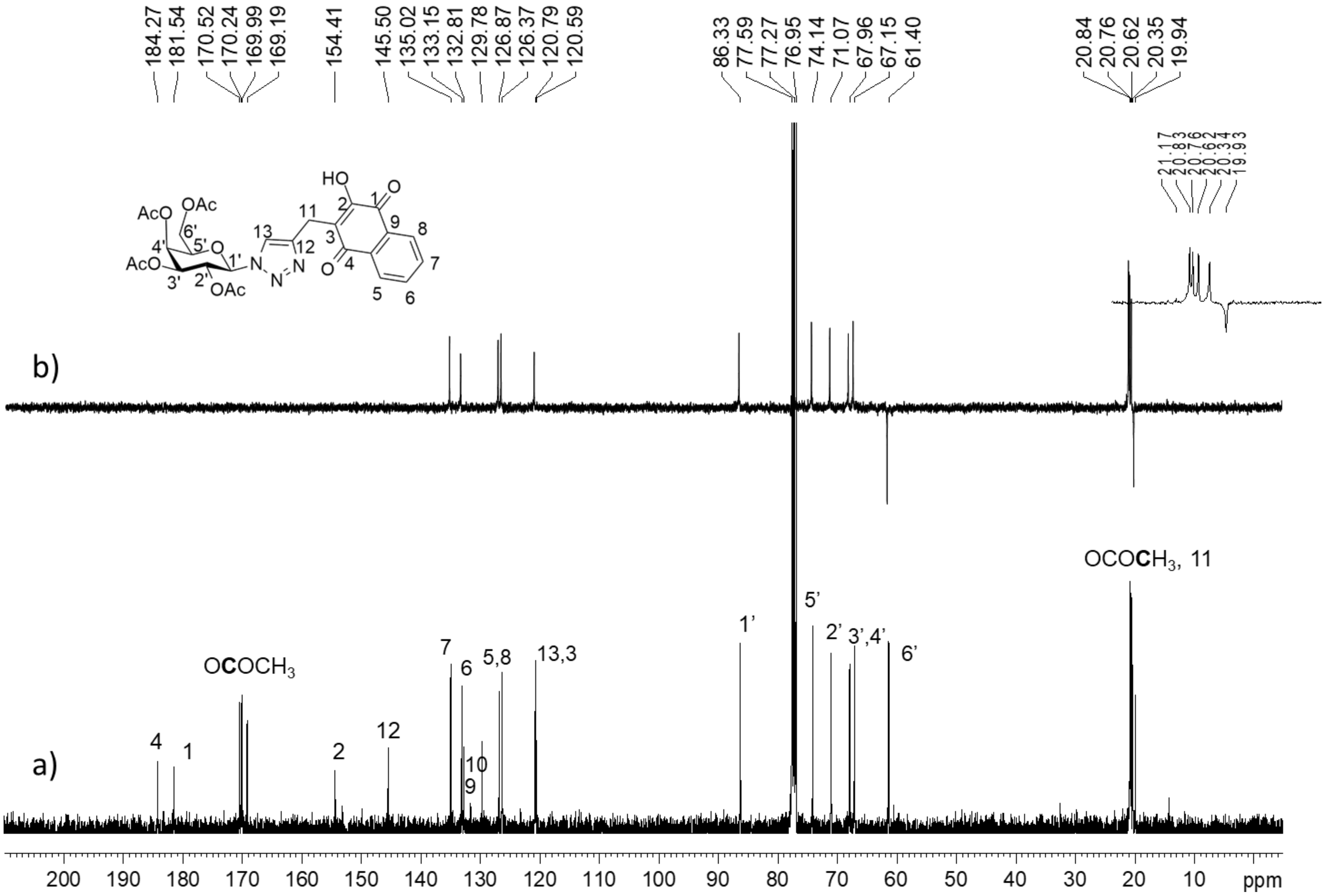

^{13}C NMR spectrum of 6 (100 MHz, CDCl3) and b) DEPT-135 of 6.

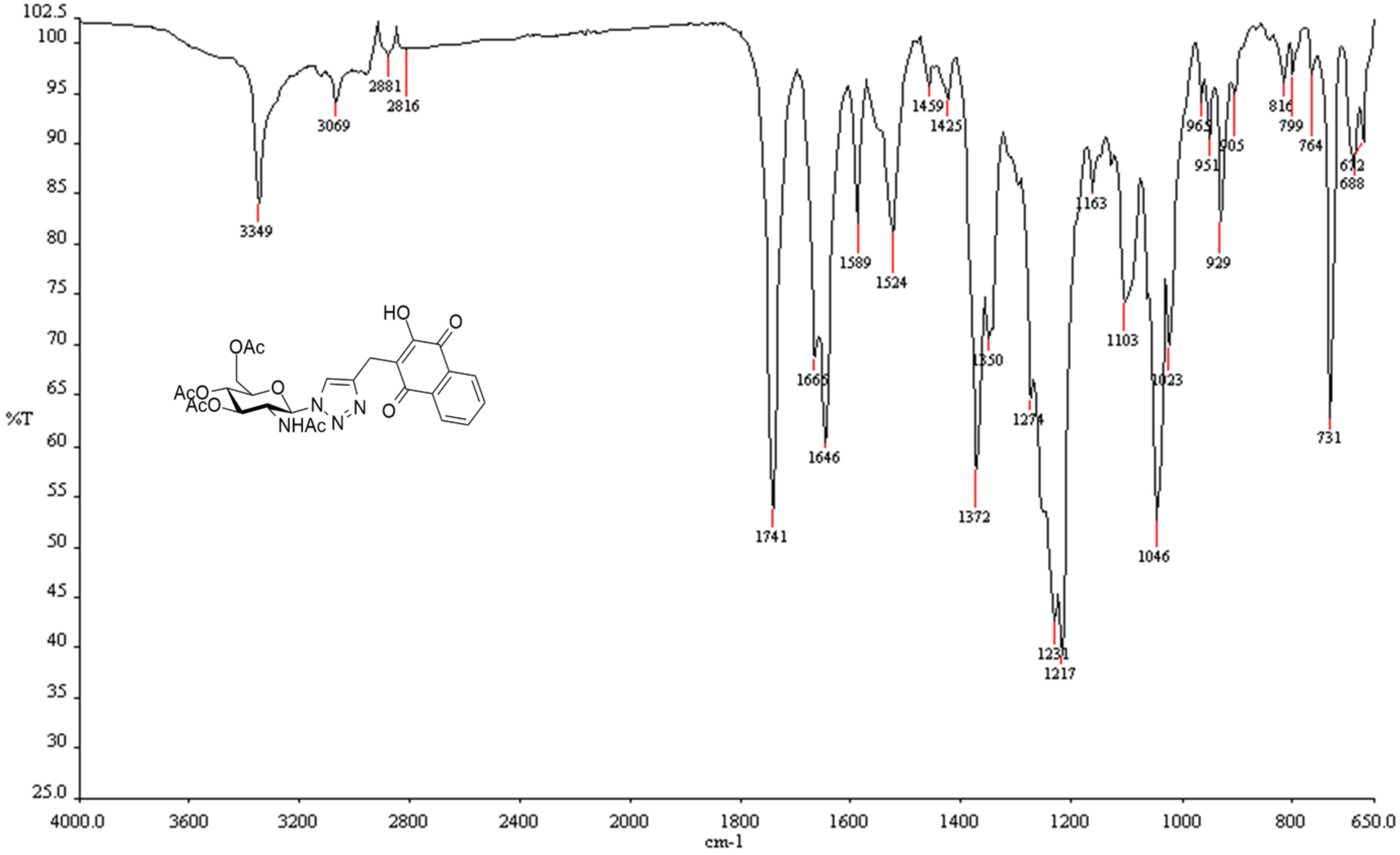

Infrared spectrum of 7

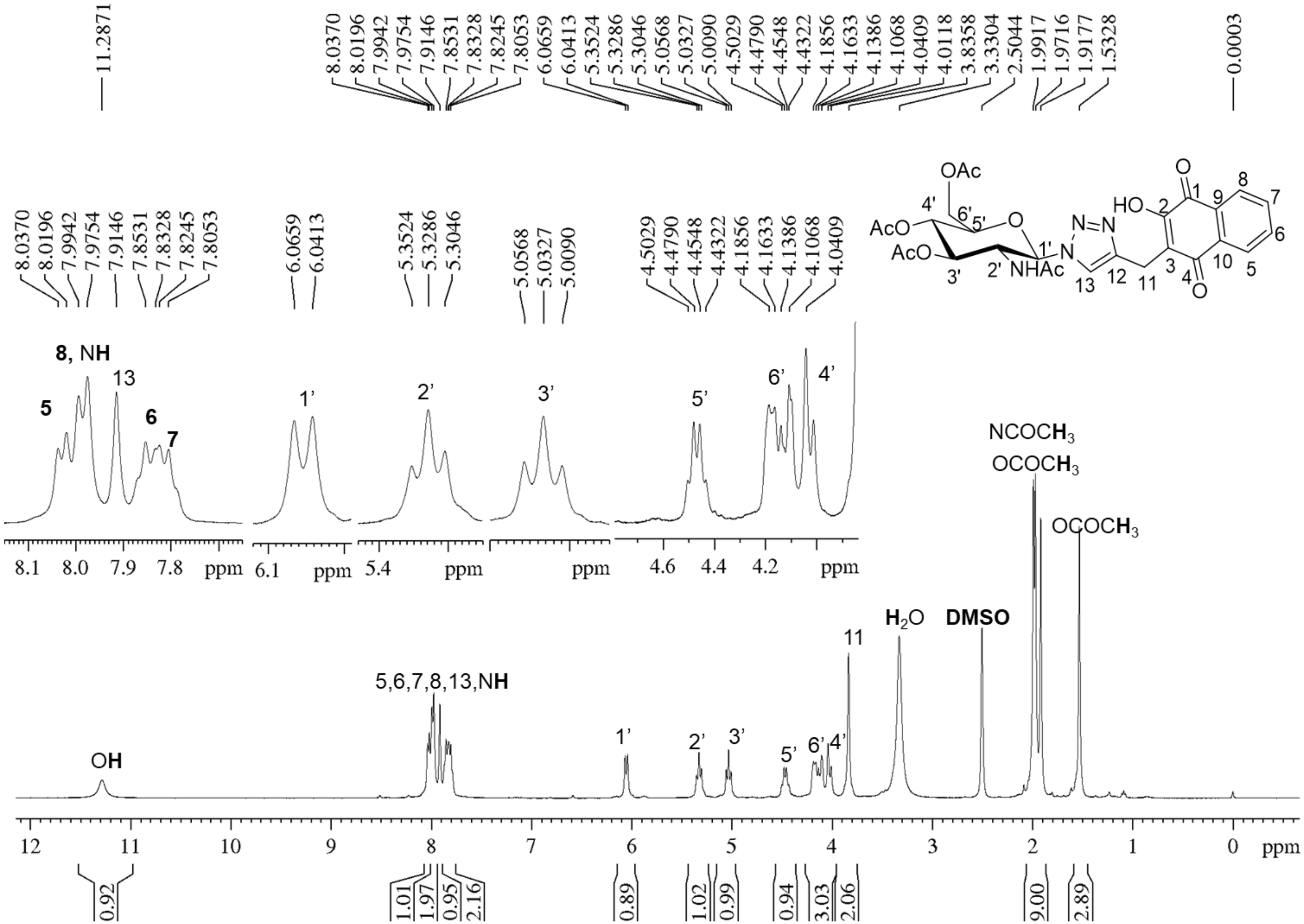

^{1}H NMR spectrum of 7 (400 MHz, DMSO-d6)

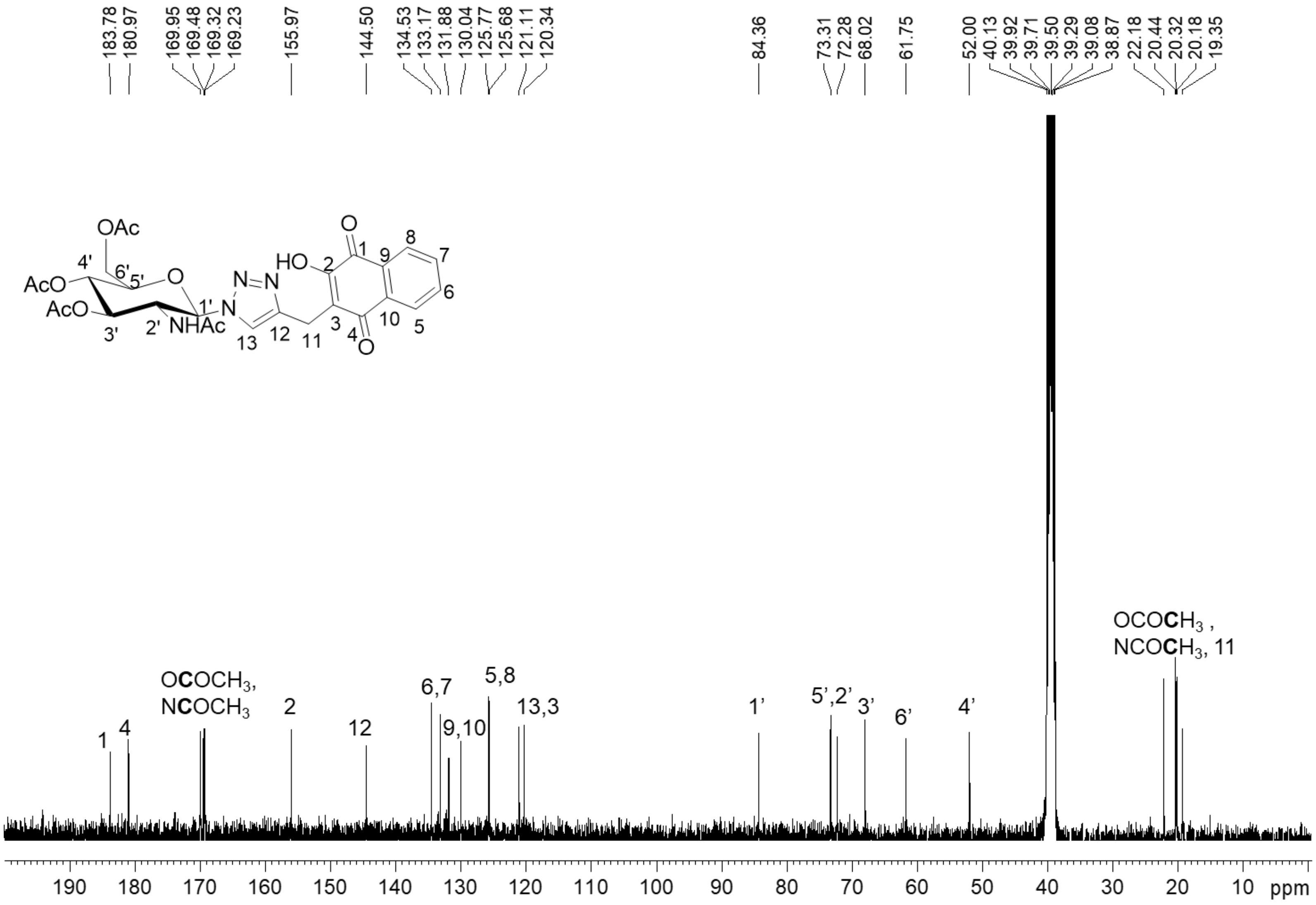

^{13}C NMR spectrum of 7 (100 MHz, DMSO-d_6)

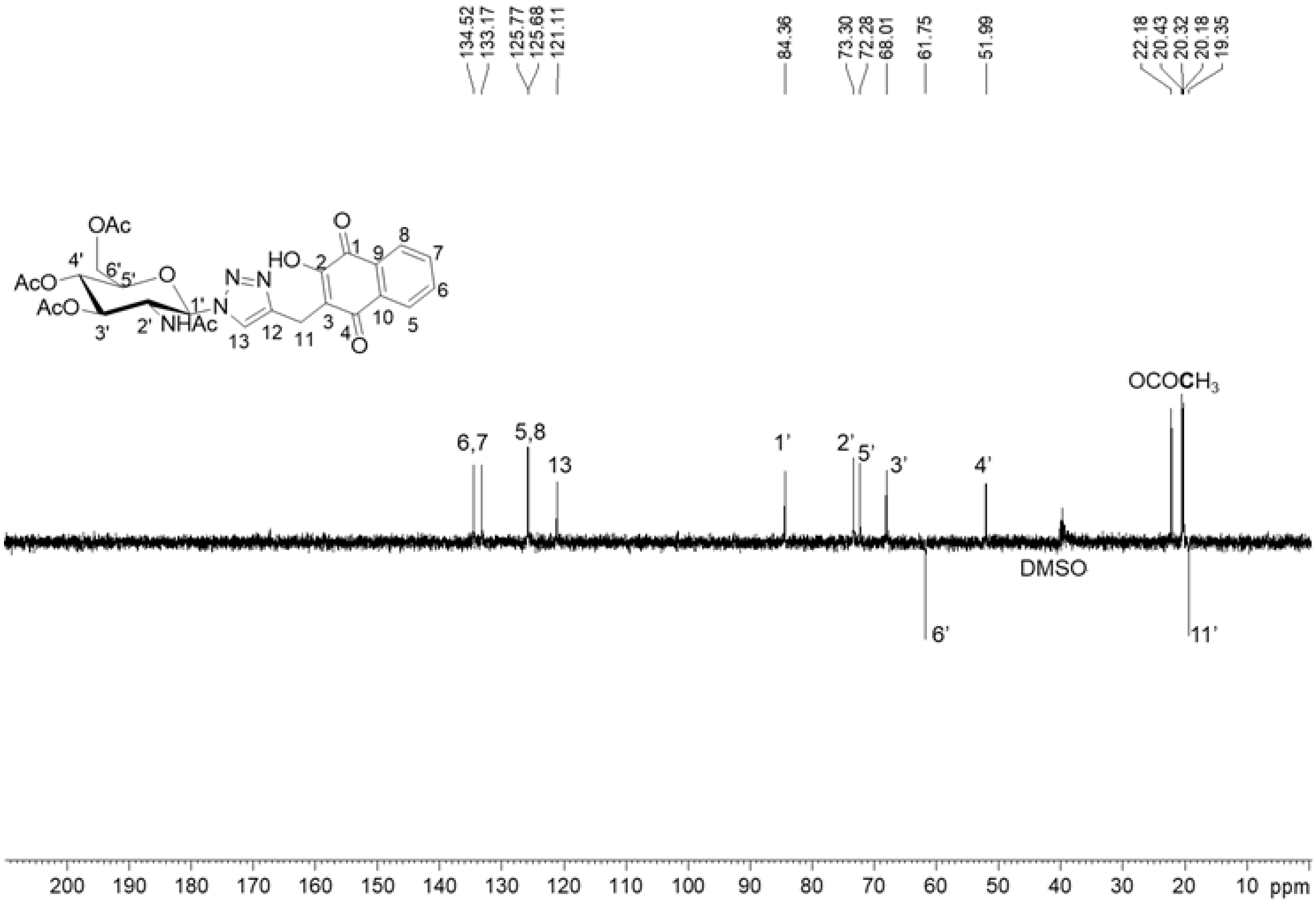

EPT-135 of 7 (DMSO-d$_6$)

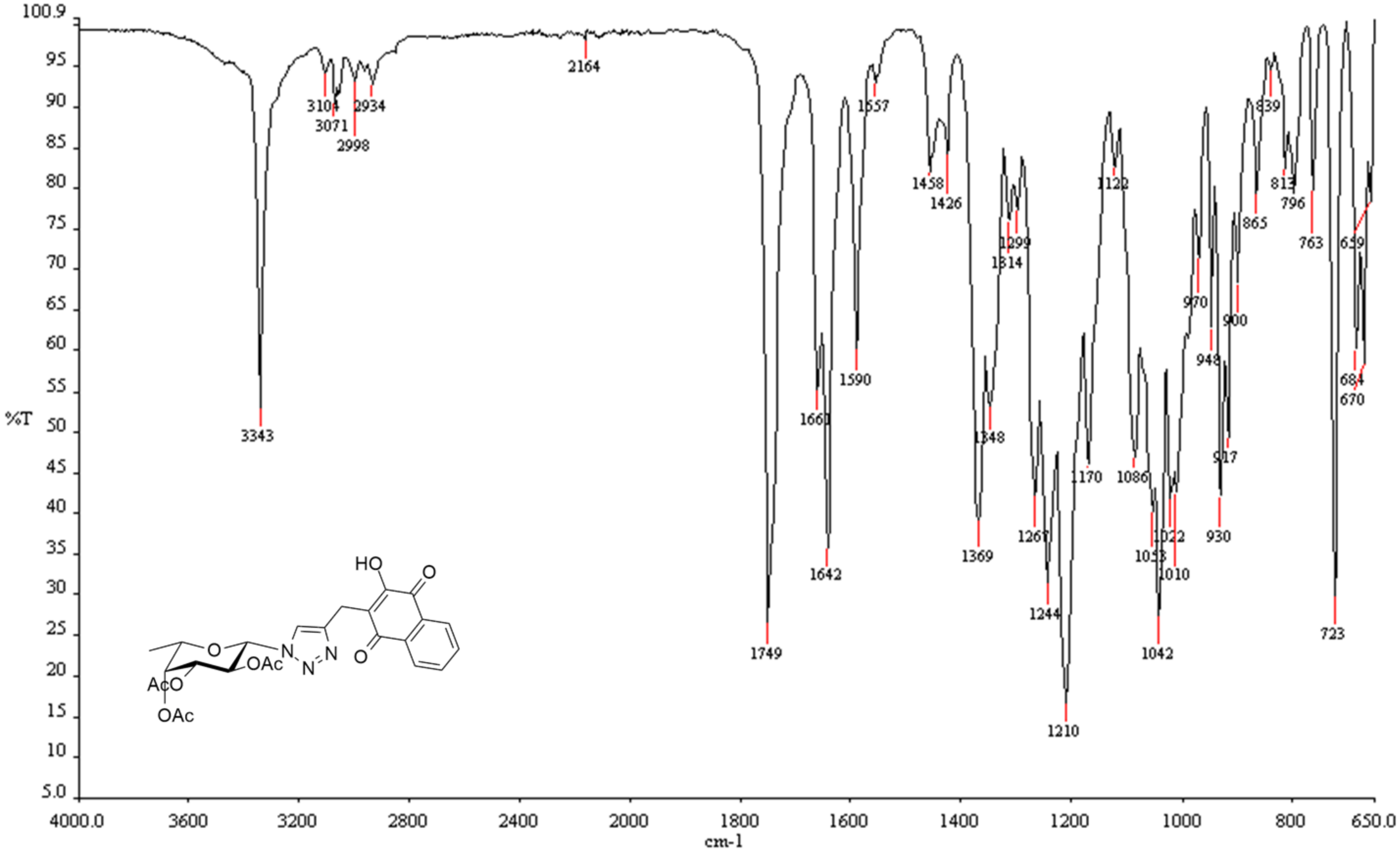

Infrared spectrum of 8

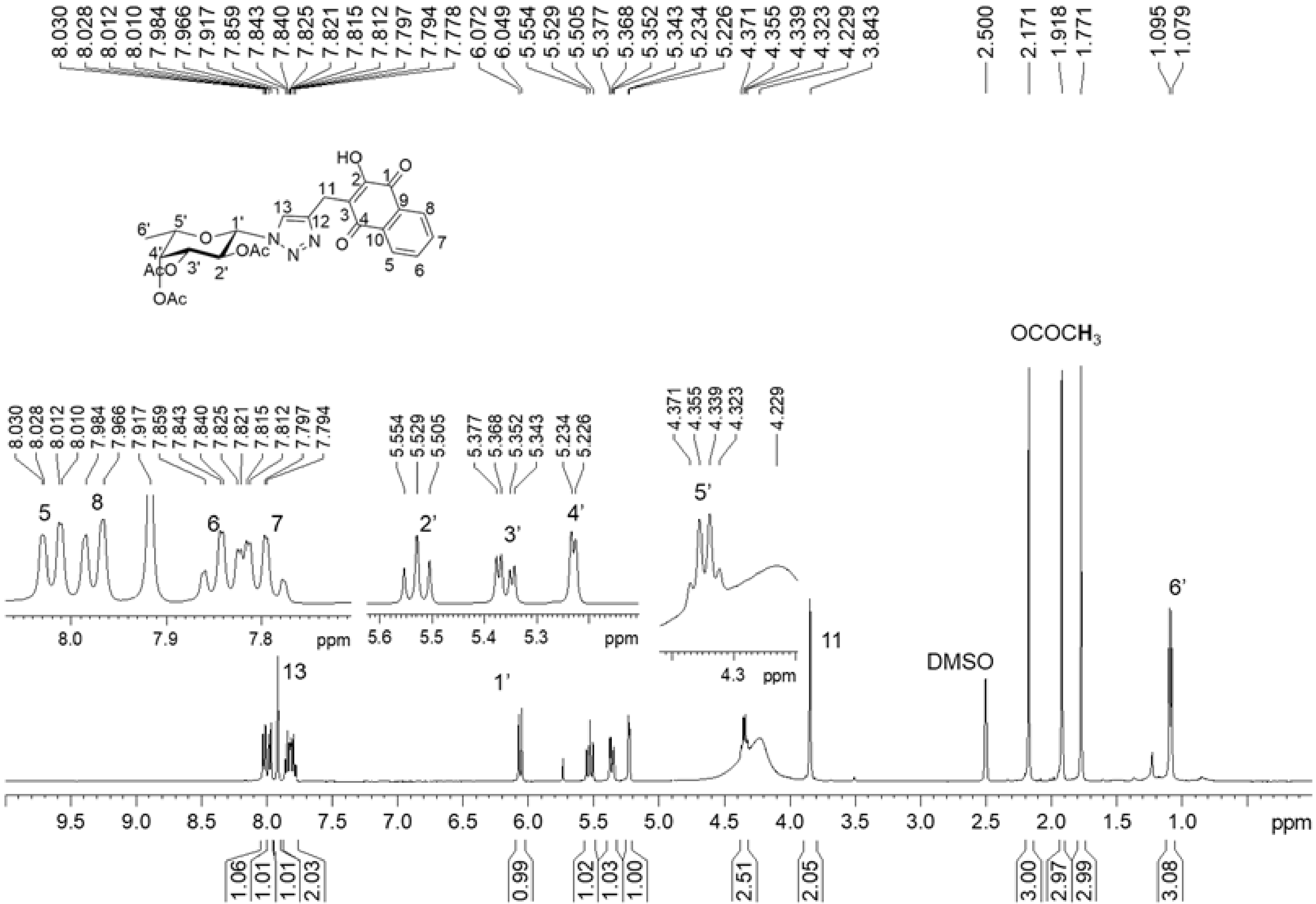

^{1}H NMR spectrum of 8 (400 MHz, DMSO-d6)

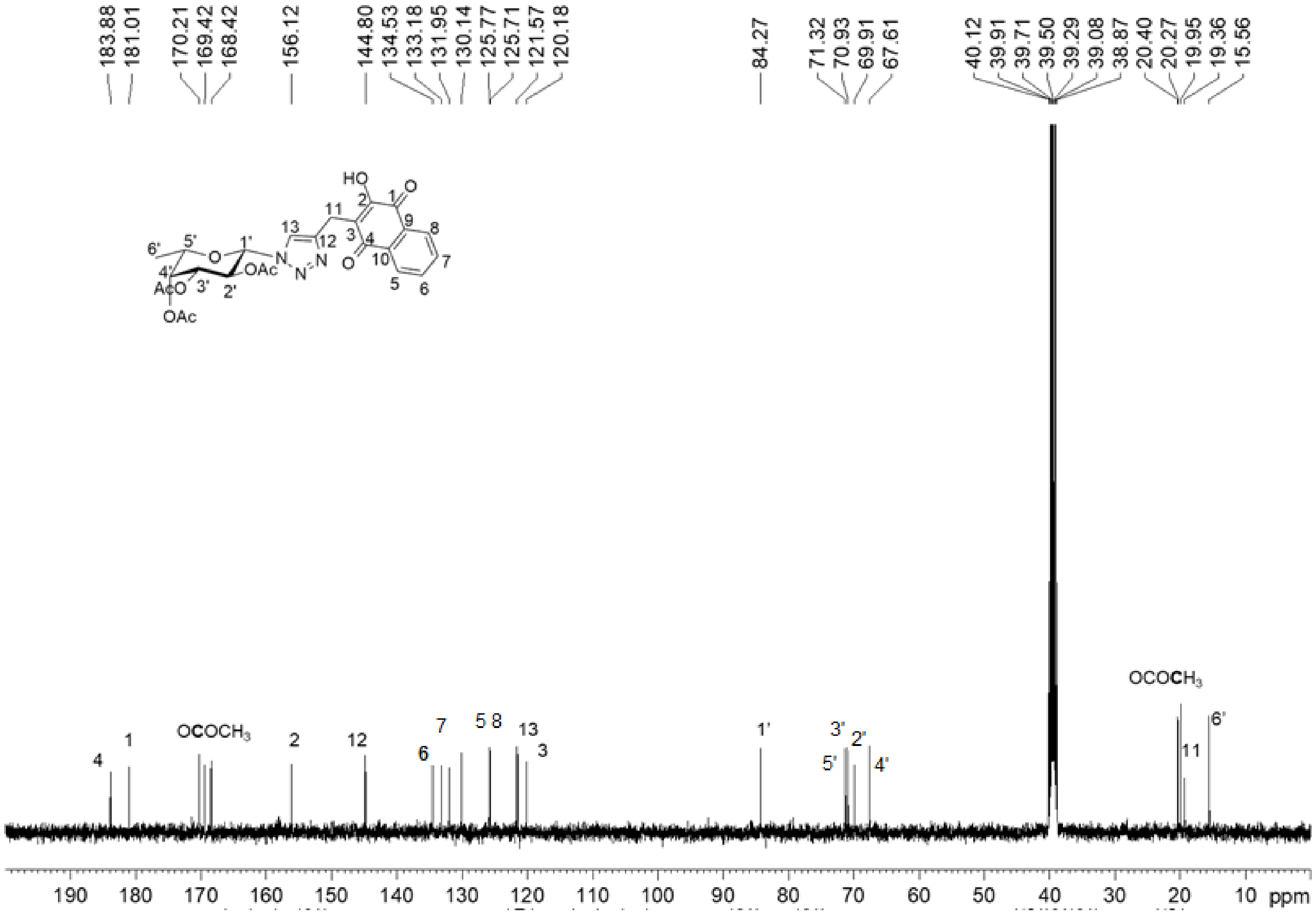

^{13}C NMR spectrum of 8 (100 MHz, DMSO-d$_6$)

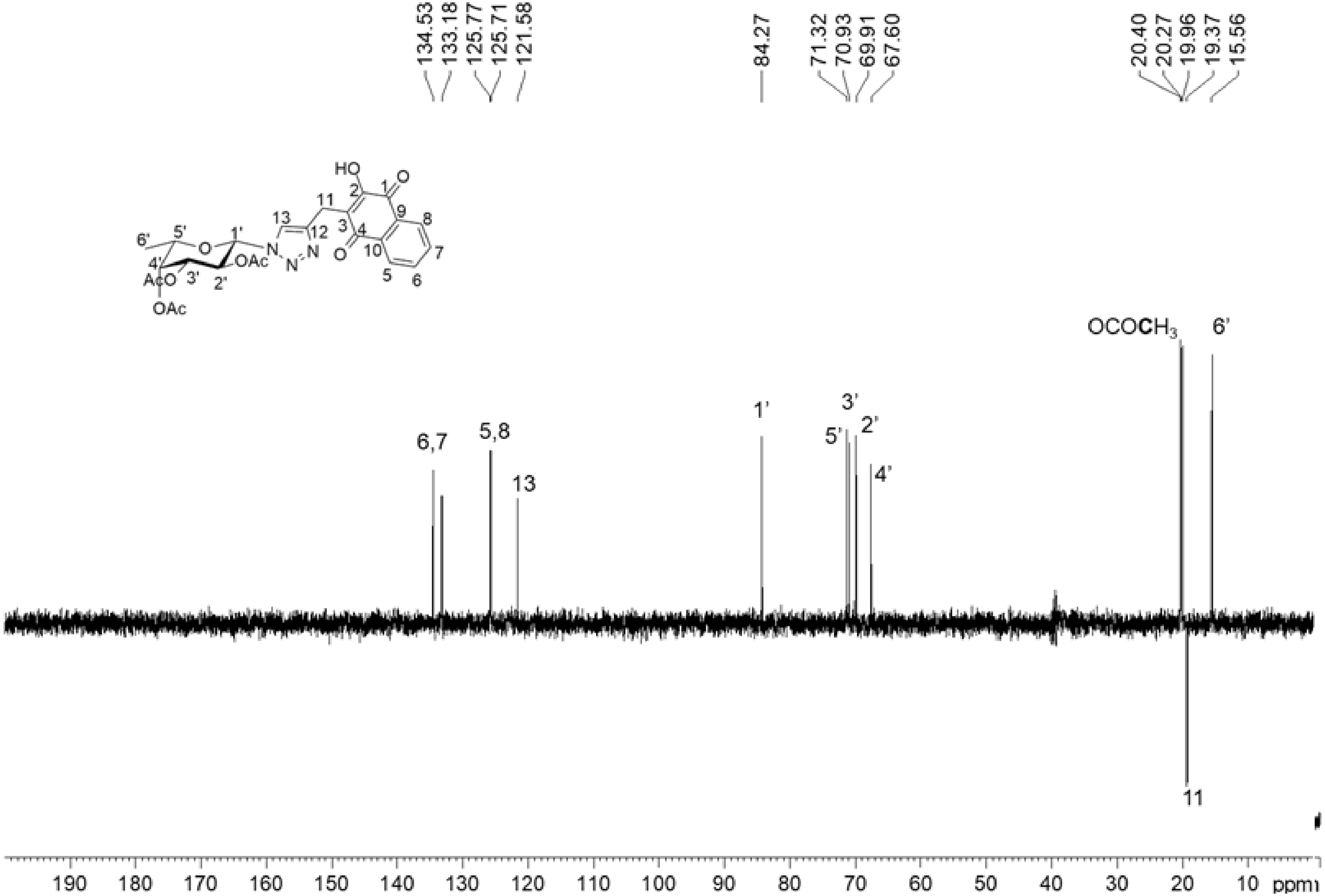

DEPT-135 of 8 (DMSO-d$_6$)
